# Optogenetically-induced multimerization of the dopamine transporter increases uptake and trafficking to the plasma membrane

**DOI:** 10.1016/j.jbc.2021.100787

**Published:** 2021-05-18

**Authors:** Shalonda M. Ingram, Tanu Rana, Ashley M. Manson, Faisal M. Yayah, Evan G.B. Jackson, Christopher Anderson, Benem-Orom Davids, J. Shawn Goodwin

**Affiliations:** Department of Biochemistry, Cancer Biology, Neuroscience, and Pharmacology, School of Medicine, Meharry Medical College, Nashville, Tennessee, USA

**Keywords:** dopamine transporter (DAT), oligomer, multimer, optogenetics, cryptochrome 2 (Cry2), methamphetamine (METH), AMPH, amphetamine, Baf A1, bafilomycin A1, Cal C, calphostin C, CD, cytochalasin D, COC, cocaine, Cry2, cryptochrome 2, Cry2–DAT, cryptochrome 2 and DAT with an mCherry fluorescent reporter, D, diffusion coefficient, DA, dopamine, DAT, dopamine transporter, FBS, fetal bovine serum, FRAP, fluorescence recovery after photobleaching, LY, LY333531, mCh, mCherry, METH, methamphetamine, M_f_, mobile fraction, NA, numerical aperture, NOM, nomifensine, PI, protease inhibitor, PMA, phorbol 12-mristate 13-acetate, ROI, region of interest

## Abstract

The dopamine transporter (DAT) is essential for the reuptake of the released neurotransmitter dopamine (DA) in the brain. Psychostimulants, methamphetamine and cocaine, have been reported to induce the formation of DAT multimeric complexes *in vivo* and *in vitro*. The interpretation of DAT multimer function has been primarily in the context of compounds that induce structural and functional modifications of the DAT, complicating the understanding of the significance of DAT multimers. To examine multimerization in the absence of DAT ligands as well as in their presence, we developed a novel, optogenetic fusion chimera of cryptochrome 2 and DAT with an mCherry fluorescent reporter (Cry2–DAT). Using blue light to induce Cry2–DAT multimeric protein complex formation, we were able to simultaneously test the functional contributions of DAT multimerization in the absence or presence of substrates or inhibitors with high spatiotemporal precision. We found that blue light–stimulated Cry2–DAT multimers significantly increased IDT307 uptake and MFZ 9-18 binding in the absence of ligands as well as after methamphetamine and nomifensine treatment. Blue light–induced Cry2–DAT multimerization increased colocalization with recycling endosomal marker Rab11 and had decreased presence in Rab5-positive early endosomes and Rab7-positive late endosomes. Our data suggest that the increased uptake and binding results from induced and rapid trafficking of DAT multimers to the plasma membrane. Our data suggest that DAT multimers may function to help maintain DA homeostasis.

The dopamine transporter (DAT) is a presynaptic plasma membrane (PM) protein responsible for terminating dopamine (DA) neurotransmission and maintaining DA homeostasis in the brain (1). Psychostimulants such as methamphetamine (METH) and cocaine (COC) target the DAT and disrupt the clearance of released DA (2, 3). METH is a substrate for the DAT and induces reverse transport of DA through the transporter (4, 5). COC binds to the outward-facing conformation of the DAT and blocks the reuptake of DA (6). The disruption of DA clearance through DAT is a primary mechanism underlying psychostimulant addiction (7).

In response to psychostimulants, the DAT forms heteromultimeric and homomultimeric complexes regulating its trafficking and function (8, 9, 10). DAT multimerization is not required for DA uptake but allows for efficient trafficking of newly synthesized DATs from the endoplasmic reticulum to the PM (8). At the cell surface, under basal conditions, the DAT exists primarily as monomers and dimers (11). DAT dimers and higher order multimers are not easily detected with Western blot unless cross-linked, suggesting relatively low-affinity interactions (11, 12). METH and AIM-100, an Ack1 inhibitor, have been shown to induce higher order DAT multimers, and both compounds increase DAT trafficking away from the PM, decreasing DAT uptake function (13, 14). Previous reports indicate that COC induces a DAT tetramer and promotes DAT trafficking to the PM, although there is no evidence that COC-induced multimers are responsible for the increased PM expression of the DAT (14, 15). Interestingly, COC-induced DAT multimers can be dissociated upon the binding of DAT substrates DA and amphetamine (AMPH) (16, 17). Moreover, a recent study has linked AMPH dispersion of COC-induced multimers to reduced COC intake in male rats (18).

Although there are numerous reports of DAT multimer formation, the functional interpretation of DAT multimerization remains unclear and has been limited by relying on mutations of the protein, compounds which cross-link the transporter at the PM or compounds that bind to the active transport pocket or allosteric site on the DAT (4, 7, 8, 9, 10, 12, 14, 19). To overcome these obstacles, we developed a novel, optogenetic, ligand-independent construct to allow us to test the functional importance of DAT multimer formation with high spatiotemporal precision. The *Arabidopsis* flavoprotein cryptochrome 2 (Cry2) is an optogenetic protein forming homomultimers upon blue light absorption and has been used to probe for the functional significance of multimerization of proteins (20, 21, 22, 23, 24, 25, 26, 27, 28). If photostimulation is not maintained, the induced multimers naturally revert to the preactivated state, providing a tightly controlled reversible model to probe cellular functions (29, 30).

We created an optogenetic fusion chimera of cryptochrome 2 and DAT with an mCherry (mCh) fluorescent reporter (referred to as Cry2–DAT), to finely control DAT multimeric protein complex formation to test the functional importance of DAT multimerization in both the absence and presence of ligands for the DAT. This model provided a means to test functional contributions of DAT multimerization in the absence of chemical modulation or structural manipulation, while preserving spatiotemporal dynamics (31). We found that when stimulated with blue light alone, Cry2–DAT rapidly multimerized and induced a significant and robust increase in IDT307 uptake and MFZ 9 to 18 binding. Furthermore, decreases in uptake and binding due to pretreatment with METH or nomifensine (NOM) were recovered when Cry2–DAT multimerization was stimulated by blue light. Our data suggest that the robust increases in uptake and binding after stimulating DAT multimerization are due to upregulation of DAT trafficking through endocytic recycling pathways. We propose that DAT multimers are not formed as a direct result of binding ligands, nor induce downregulation at the PM, but that DAT multimerization induces rapid trafficking of the naïve DAT to the PM to facilitate DA uptake. To our knowledge, this is the first study to use an optogenetic approach to directly test the consequences of DAT multimerization in the absence of compounds that bind to DAT.

## Results

### The novel Cry2–DAT optogenetic construct encodes a DAT with normal cell surface expression and uptake activity

**Figure 1:**
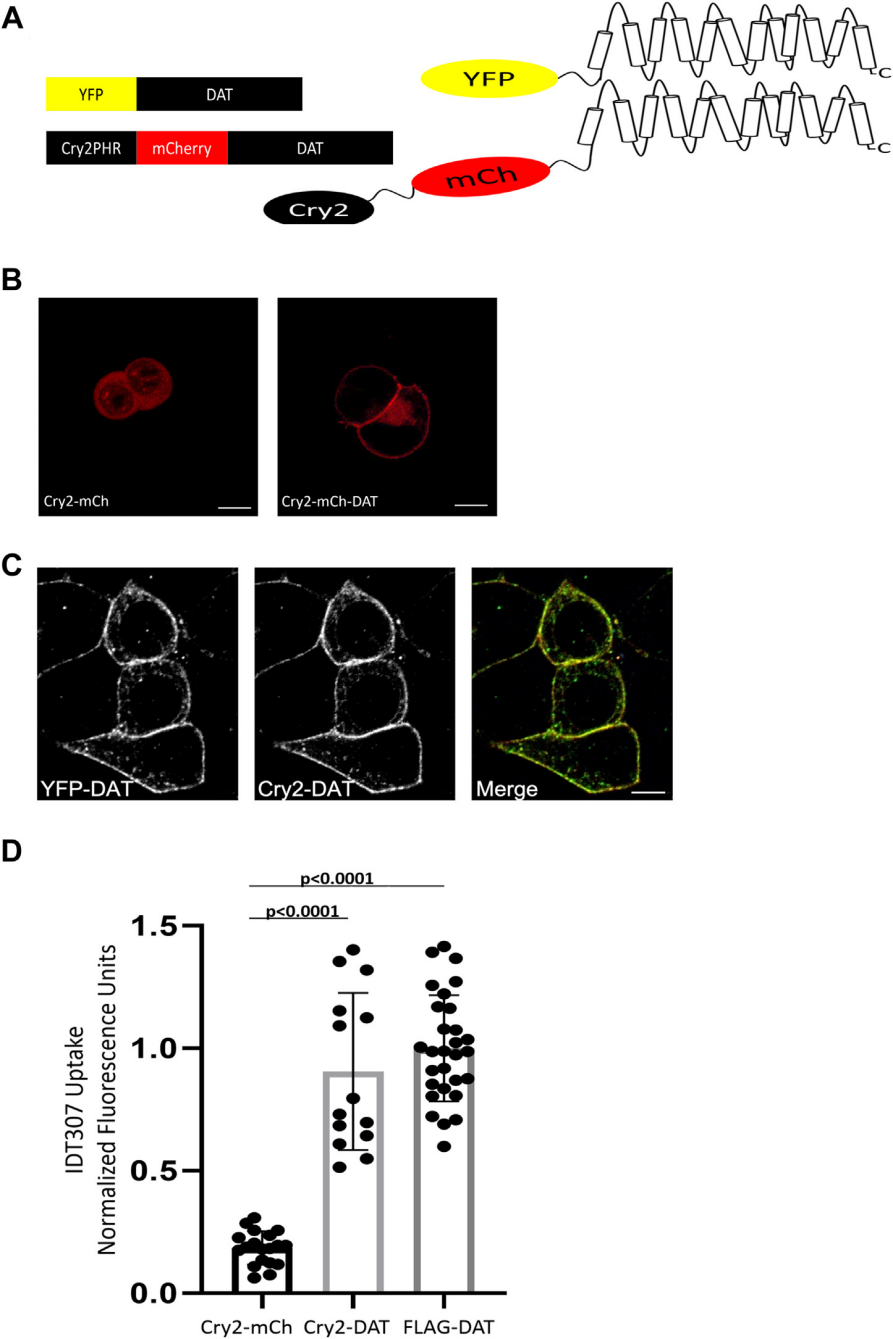
**The Novel Cry2–DAT optogenetic construct encodes a DAT with normal cell-surface expression and uptake activity.***A*, YFP–DAT and Cry2–DAT construct maps display the DAT location in respect to YFP or Cry2PHR–mCh. The cartoons to the right of the construct maps demonstrate the N-terminal location of the YFP and Cry2–mCherry. For the Cry2–DAT construct, the location of mCherry is the same as YFP, whereas Cry2 is N-terminal to mCherry. *B*, confocal images of live HEK293 cells expressing the Cry2–mCh empty vector show a cytoplasmic localization. Live-cell confocal imaging of HEK293 cells expressing Cry2–DAT localizes primarily to the plasma membrane. *C*, YFP–DAT and Cry2–DAT coexpressing HEK293 cells show strong colocalization with a Pearson’s correlation coefficient of 0.87. *D*, the Cry2–mCh vector, FLAG–DAT, or Cry2–DAT was expressed in HEK293 cells before imaging IDT307 uptake. The Cry2–mCh empty vector has minimal uptake of IDT307. Cry2–DAT and FLAG–DAT have the same uptake capacity for IDT307 (*p* < 0.0001, n = 19). Significance was determined using one-way ANOVA with Tukey’s multiple comparisons test. The scale bar represents 5 μm. Cry2, cryptochrome 2; DAT, dopamine transporter; mCh, mCherry.

To construct Cry2–DAT, the DAT coding sequence was inserted into the Cry2–mCh vector to express Cry2–mCh at the N terminus of the DAT (Fig. 1*A*) in the same manner as YFP–DAT and FLAG DAT because these DAT chimeras with N-terminal YFP and FLAG tags resulted in normal DAT function (9, 16, 32, 33, 34, 35, 36). Next, we expressed Cry2–DAT or Cry2–mCh empty vector in HEK293 cells to test the localization of these constructs in the cell. Cry2–DAT was expressed at the PM, while Cry2–mCh expression was cytoplasmic (Fig. 1*B*). Coexpression of Cry2–DAT and YFP–DAT showed strong colocalization (Pearson’s correlation coefficient of 0.87), particularly at the PM (Fig. 1*C*).

To ensure the Cry2–DAT construct encoded a DAT protein with normal function, the uptake activities of FLAG–DAT and Cry2–DAT were tested. Cry2–DAT–expressing cells had uptake activity comparable with FLAG–DAT–expressing cells, and cells expressing the DAT construct had significantly more uptake than cells expressing the vector alone (Fig. 1*D*). These data show that Cry2–DAT has expression and function comparable with YFP–DAT and FLAG–DAT.

### Light-induced Cry2–DAT multimers mimic METH-induced YFP–DAT multimers

**Figure 2:**
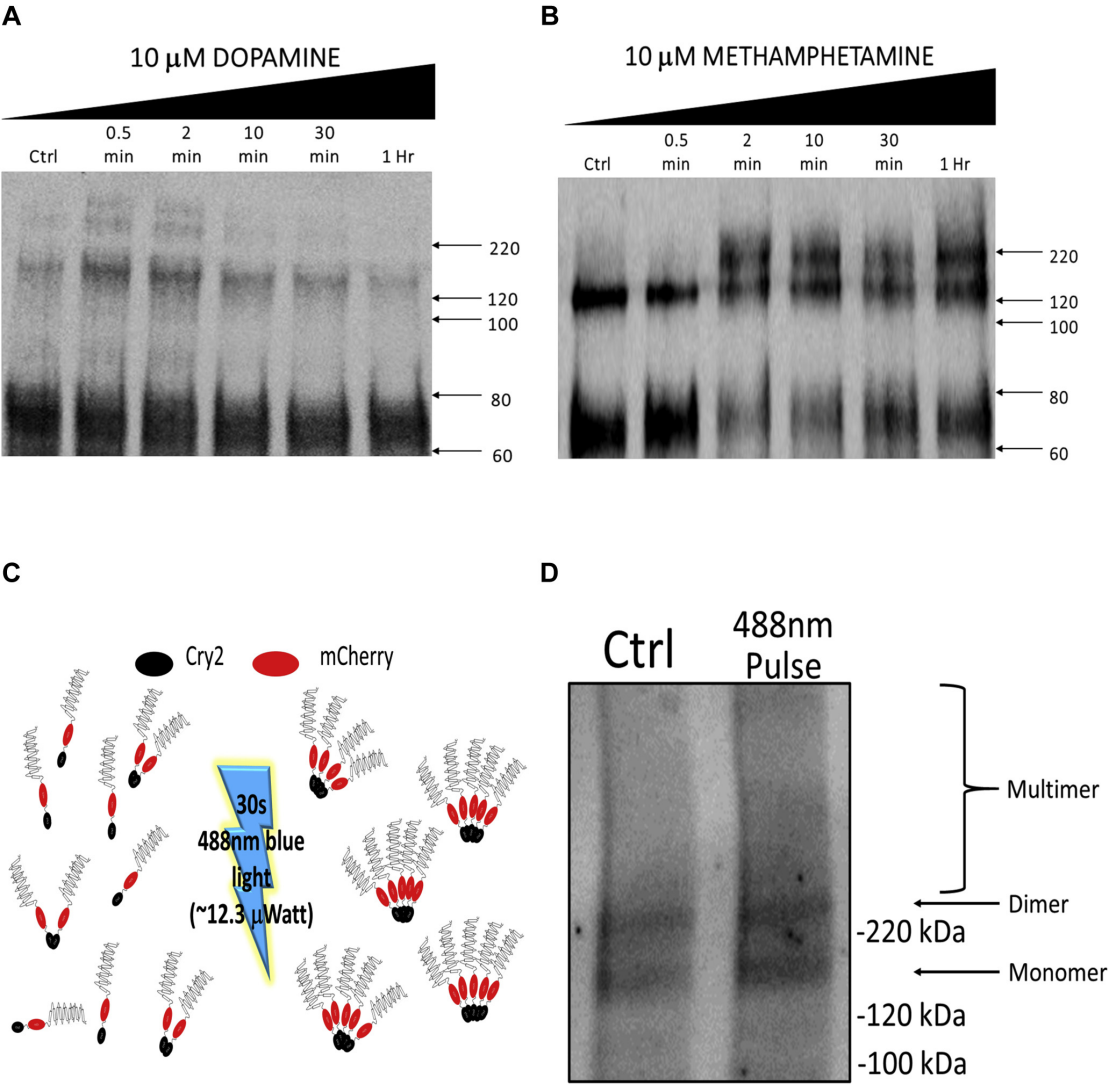
**Light-induced Cry2–DAT multimers mimic DA- and METH-induced YFP–DAT multimers.***A*, HEK cells stably expressing YFP–DAT were treated with 10 μM DA or (*B*) 10 μM METH for the indicated times. After DA or METH treatment, CuP was used to crosslink the DAT to retain multimer bands. The Mem-PER Plus Membrane Protein Extraction kit was used to isolate the plasma membrane DAT. Membrane fractions were run on an 8% polyacrylamide gel. Data shown in *panels A* and *B* are representative Western blots showing time-dependent formation of DAT multimers from treatment with DA or METH. *C*, HEK cells were transfected with Cry2–DAT and incubated in the dark (control) or exposed to *blue light* for 30 s. Cry2–DAT biotinylated samples were then run on a 4–15% gradient polyacrylamide gel. *D,* a representative Western blot of cell surface biotinylation of Cry2–DAT with and without *blue light* exposure shows DAT multimer formation after exposure to *blue light* for 30 s. CuP, copper phenanthroline; Cry2, cryptochrome 2; Cry2–DAT, cryptochrome 2 and DAT with an mCherry fluorescent reporter; DA, dopamine; DAT, dopamine transporter; METH, methamphetamine.

A baseline time course for YFP–DAT multimer formation was established for the DAT substrates, DA and METH, using the cross-linking agent copper phenanthroline and membrane enrichment to detect DAT multimer formation (15, 37). DAT dimers were detected under control conditions, as previously reported (14). DA treatment induced a DAT multimer banding pattern corresponding to a tetramer after 30 s of treatment; after 2 min, the DAT tetramers dissipated to dimers (Fig. 2*A*). In contrast, treatment with METH induced DAT trimers at 2 min, and this multimer pattern was maintained up to 60 min (Fig. 2*B*).

Using biotinylation to detect cell surface Cry2–DAT (~130 kDa), monomer and dimer bands were detected for control conditions. Using blue light to stimulate Cry2–DAT multimers, we detected DAT multimer expression at the cell surface. Although the resolution of the higher order Cry2–DAT multimer pattern could not be accurately determined using biotinylation to isolate cell surface DAT, the multimers are in a MW range corresponding to higher order Cry2–DAT multimeric complexes (Fig. 2*D*).

Taken together, these data establish that in control conditions, YFP–DAT and Cry2–DAT form dimers and that blue light stimulation of Cry2–DAT allows for the detection of higher order complexes without the addition of DA or METH.

### YFP–DAT is kinetically trapped in Cry2–DAT multimers

**Figure 3:**
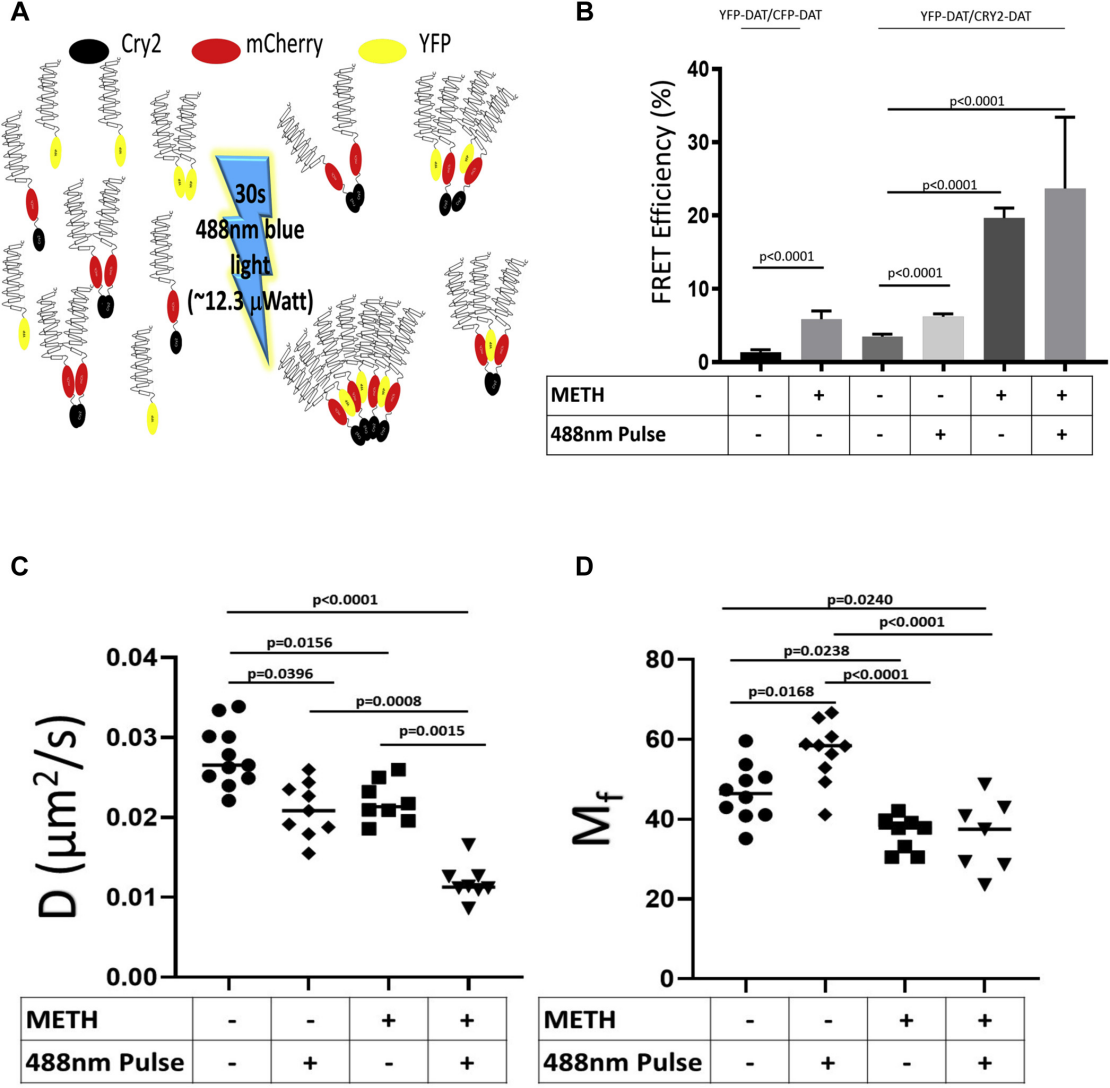
**YFP–DAT can be kinetically trapped in Cry2–DAT multimers.***A*, the schematic depicts the incorporation of YFP–DAT into Cry2–DAT multimers after *blue light* stimulation. *B*, Cry2–DAT or CFP–DAT was transfected into HEK cells stably expressing YFP–DAT. FRET for CFP–DAT and YFP–DAT was used as a basis of comparison to test if *blue light* multimerization of Cry2–DAT could kinetically trap YFP–DAT into Cry2–DAT multimers by measuring FRET efficiencies. The increases in FRET efficiencies for YFP–DAT and Cry2–DAT compared with CFP–DAT and YFP–DAT suggest that YFP–DAT is incorporated into Cry2–DAT multimers (n = 3–5, 0.001 > *p* < 0.05). One-way ANOVA with Tukey’s multiple comparisons test was applied to determine significance of data. *C* and *D*, the lateral mobility of YFP–DAT was tested in the absence or presence of Cry2–DAT to determine if Cry2–DAT multimers would incorporate YFP–DAT to impact its (*C*) diffusion coefficient (D) and (*D*) mobile fraction (M_f_). HEK cells stably expressing YFP–DAT alone or transfected with Cry2–DAT were used for FRAP. A circular ROI with a 5-μm diameter was placed at the plasma membrane for photobleaching. (n = 8–11, 0.05 > *p* < 0.001). Mixed-effects analysis with Tukey’s multiple comparison test was used to determine significance of data. Cry2, cryptochrome 2; Cry2–DAT, cryptochrome 2 and DAT with an mCherry fluorescent reporter; DAT, dopamine transporter; FRAP, fluorescence recovery after photobleaching; ROI, region of interest.

Previous studies have shown that functional optogenetic complexes can kinetically trap nonoptogenetic binding partners of the complex upon blue light stimulation, which can be detected by using FRET and fluorescence recovery after photobleaching (FRAP) (29). We tested if blue light–stimulated Cry2–DAT could kinetically trap YFP–DAT into multimer complexes using FRAP to evaluate codiffusion and FRET to assess proximity of the two DAT constructs (Fig. 3*A*). Two nonoptogenetic proteins, CFP–DAT and YFP–DAT, were used as a comparison for DAT multimerization, as previously reported (9, 18). The FRET efficiencies for CFP–DAT and YFP–DAT treated with the vehicle or 10 μM METH were similar to previously published data (36) (Fig. 3*B* and S1*A*). YFP–DAT and Cry2–DAT FRET efficiencies increased with 10 μM METH treatment and with blue light stimulation to induce Cry2–DAT multimerization. These data were similar to the FRET efficiencies of CFP–DAT and YFP–DAT with METH treatment. There were no statistical differences for the FRET efficiencies of YFP–DAT and CFP–DAT treated with METH compared with the FRET efficiencies of YFP–DAT and Cry2–DAT with METH treatment or blue light stimulation. The FRET efficiencies of YFP–DAT and Cry2–DAT in samples pretreated with METH and subsequent blue light stimulation increased robustly compared with METH or blue light stimulation alone (Fig. 3*B*).

Live-cell FRAP was performed to test the ability of Cry2–DAT to kinetically trap YFP–DAT into blue light–induced multimers. The diffusion coefficient (D) of YFP–DAT decreased (Fig. 3*C* and S1*B*) and the mobile fraction (M_f_) (Fig. 3*D* and S1*C*) increased significantly upon blue light stimulation in cells coexpressing YFP–DAT and Cry2–DAT compared with cells expressing YFP–DAT alone. The D and M_f_ for YFP–DAT in the presence of 10 μM METH in coexpressing YFP–DAT and Cry2–DAT cells also decreased significantly compared with control. METH and subsequent blue light stimulation of cells coexpressing YFP–DAT and Cry2–DAT resulted in a significant reduction in D for YFP–DAT compared with controls and to METH treatment alone, although M_f_ was unchanged (Fig. 3*D*).

The FRET and FRAP data establish that YFP–DAT is kinetically trapped into Cry2–DAT multimers induced by blue light. The incorporation of the nonoptogenetic YFP–DAT into Cry2–DAT light-induced multimers implies that the photosensitive portion of Cry2–DAT is a means to induce DAT oligomerization.

### Blue light–stimulated Cry2–DAT multimers have increased uptake activity and an outward facing conformation at the PM

**Figure 4:**
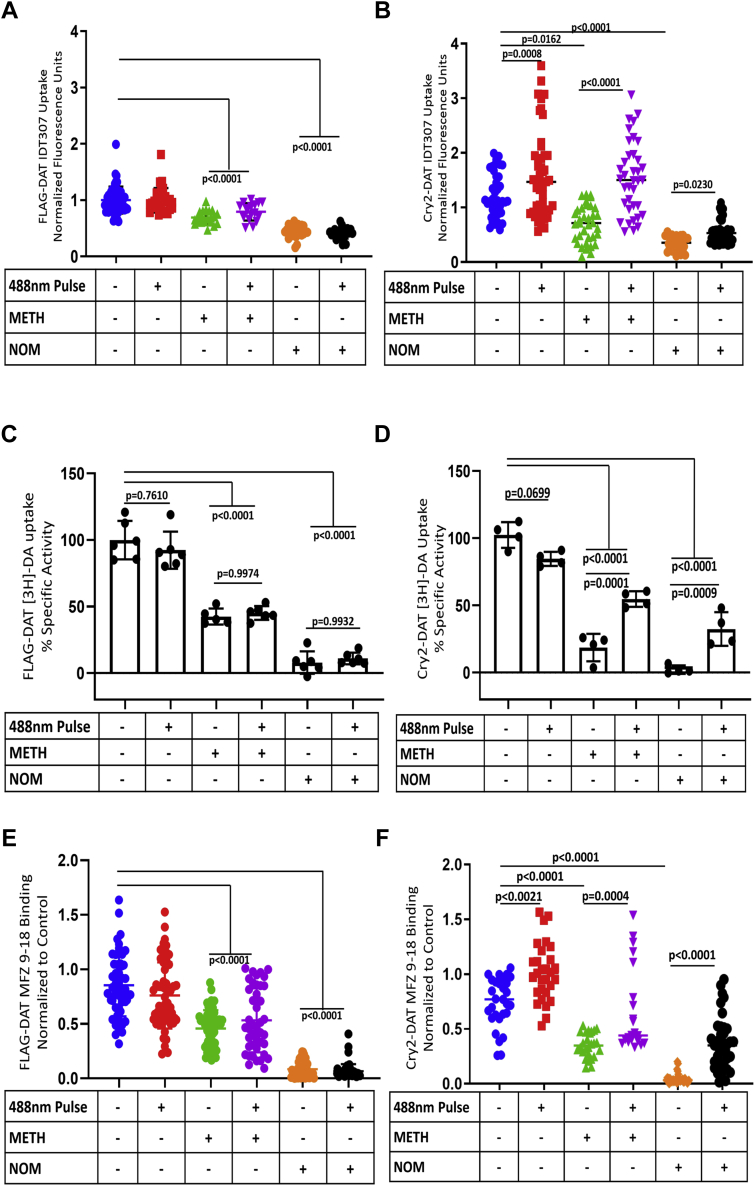
***Blue light*–stimulated Cry2–DAT multimers have increased uptake activity and an outward-facing conformation at the plasma membrane.** HEK cells stably expressing FLAG–DAT were used to as a comparison for Cry2–DAT uptake in HEK cells transiently transfected with Cry2–DAT. *A*, FLAG–DAT or (*B*) Cry2–DAT uptake of IDT307 was evaluated after pretreatment with vehicle, 10 μM METH, or 10 μM NOM. Where indicated, cells were also exposed to 488-nm light (n = 24–30, 0.05 > *p* < 0.001). *C*, FLAG–DAT or (*D*) Cry2–DAT [3H]-DA-uptake assay was evaluated after pretreatment with the vehicle, 10 μM METH, or 10 μM NOM. Where indicated, cells were also exposed to 488-nm light (n = 30–40, 0.05 > *p* < 0.001). *E*, FLAG–DAT or (*F*) Cry2–DAT binding of MFZ 9-18 was evaluated after pretreatment with vehicle, 10 μM METH, or 10 μM NOM. Where indicated, cells were also exposed to 488-nm light (n = 30–40, 0.05 > *p* < 0.001). Cry2, cryptochrome 2; Cry2–DAT, cryptochrome 2 and DAT with an mCherry fluorescent reporter; DAT, dopamine transporter; METH, methamphetamine; NOM, nomifensine.

Prior studies have shown that agents known to induce multimerization of DAT, such as METH and COC, reduce the uptake of DA (3, 38, 39, 40). We tested the effects of METH, a DAT substrate, and NOM, a DAT blocker, to evaluate how blue light–induced multimerization may affect the uptake of IDT307 or [^3^H]-DA and MFZ 9-18 binding in FLAG–DAT– and Cry2–DAT–expressing cells (Fig. 4). For experiments testing the effects of METH or NOM, we pretreated for 2 min because this timepoint will avoid significant downregulation of DAT induced by METH (41), allow for METH-induced DAT phosphorylation, yet achieve detectable multimerization (Fig. 2*B*), and occur within the 5.5 min half-life of Cry2 multimers (29, 30). To measure DAT uptake, we used [^3^H]-DA as done in the study by Hong and Amara (42) and IDT307 4-(4-(dimethylamino)phenyl)-1-methylpyridinium iodide (APP^+^), a fluorescent analog of 1-methyl-4-phenylpyridinium (MPP+), to measure transport activity in single cells transiently expressing Cry2–DAT using fluorescence microscopy (43).

The blue light stimulation was done at 4% of the 488-nm laser power (12.5–13 μW) immediately before performing the uptake experiment. After the addition of IDT307, uptake was assessed using 1.5% of the 488-nm laser power. Continuous monitoring of IDT307 using 1.5% of the 488-nm laser power did not affect Cry2–DAT multimerization or the uptake measurements (data not shown).

HEK293 cells expressing Cry2–mCh empty vector had minimal to no uptake activity as measured using IDT307 (Fig. 1*D*). FLAG–DAT was used as a positive control for uptake activity, but as a negative control for blue light–induced multimerization of DAT. FLAG–DAT–expressing cells treated with METH and NOM had significantly reduced uptake compared with the control cells (Fig. 4, *A* and *C*). The uptake activity of Cry2–DAT was comparable with that of FLAG–DAT, under control conditions (Fig. 4, *B* and *D* and S2). When cells were stimulated with 4% total laser output of 488-nm blue light for 30 s to induce Cry2–DAT multimers, the IDT307 uptake activity of Cry2–DAT increased significantly under control conditions, whereas no change in FLAG–DAT uptake was detected using the same blue light–stimulation protocol (Fig. 4, *A* and *B*). Treatment with METH and NOM significantly decreased Cry2–DAT IDT307 and [^3^H]-DA uptake, but surprisingly, blue light stimulation after METH and NOM treatment significantly increased uptake activity without altering the uptake for FLAG–DAT cells with the same treatment paradigm (Fig. 4, *A*–*D*). Because IDT307 and [^3^H]-DA uptake data trends were consistent, IDT307 was used preferentially to measure single cell, unidirectional uptake activity in cells expressing Cry2–DAT.

A fluorescent COC analogue, MFZ 9-18, was used to quantitatively assess the availability of Cry2–DAT in the outward-facing conformation at the PM (44). MFZ 9-18 binding assays were performed at 4 °C to minimize trafficking of the DAT during MFZ 9-18 binding. MFZ 9-18 binding to Cry2–DAT–expressing cells displayed similar trends to IDT307 uptake (Fig. 4, *E* and *F*). Pretreating Cry2–DAT cells with METH or NOM significantly decreased MFZ 9-18 binding compared with controls, as it did for the FLAG–DAT cells. Blue light stimulation of Cry2–DAT–expressing cells, after METH and NOM treatment, significantly increased binding compared with no blue light stimulation. In contrast, blue light stimulation did not change the binding of MFZ 9-18 in FLAG–DAT cells (Fig. 4*E*).

### Blue light–stimulated multimerization of Cry2–DAT induces rapid trafficking to the PM

**Figure 5:**
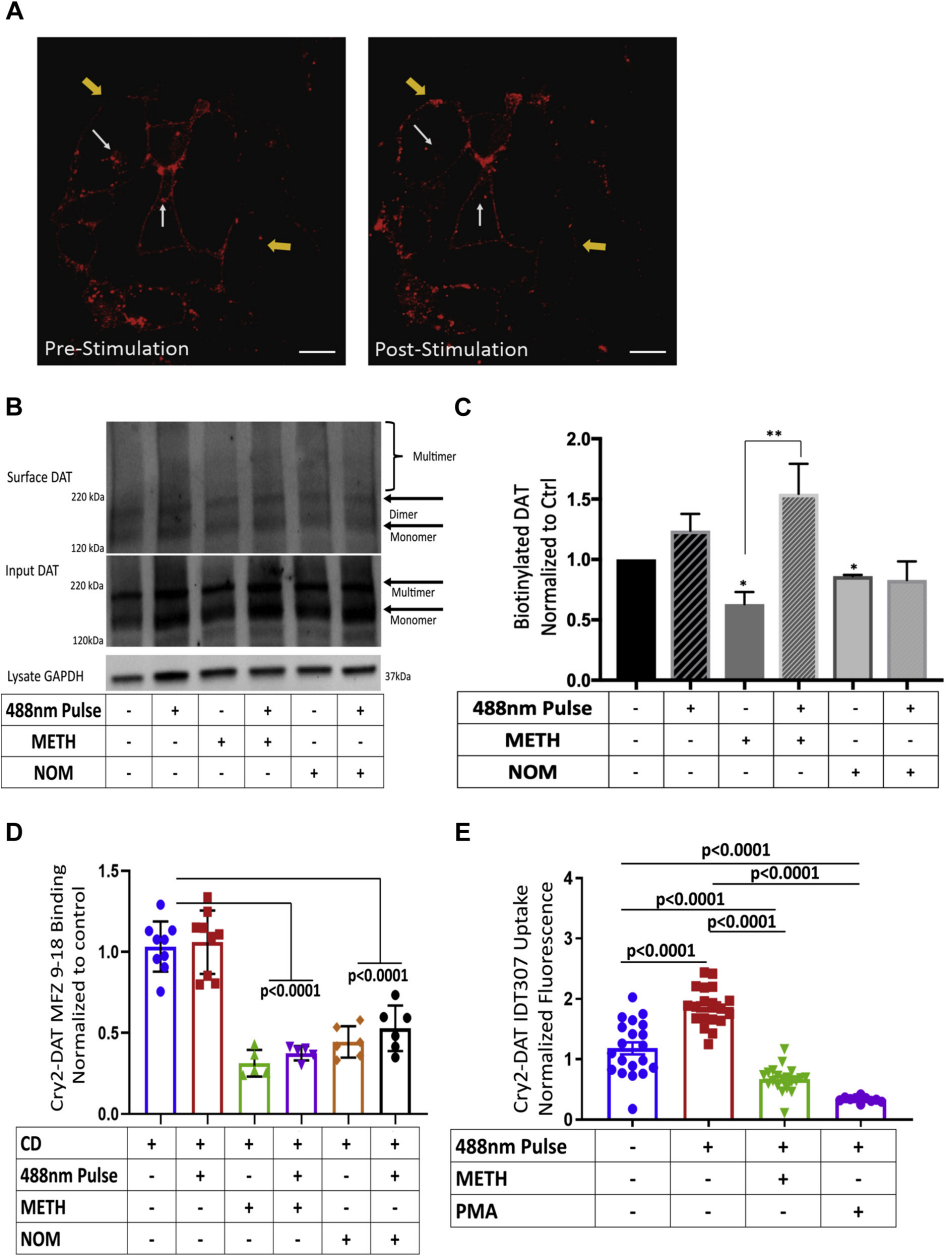
***Blue light*-stimulated multimerization of Cry2–DAT induces rapid trafficking to the plasma membrane.***A*, representative images from live confocal time lapse imaging before and after 488-nm light pulse for 30 s. After the 488-nm light pulse, Cry2–DAT showed an increased membrane expression (*yellow arrows*) and reduced in intracellular pools of Cry2–DAT (*white arrows*). *B*, representative cell-surface biotinylation Western blot of Cry2–DAT–expressing HEK cells. *C*, analysis of immunoreactivity of biotinylated Cry2–DAT Western blots in *panel B* show a statistically significant decrease in the cell-surface DAT after treatment with 10 μM METH or 10 μM NOM. A statistically significant increase in the cell-surface DAT was detected after the 488-nm light pulse and METH treatment (n = 3, ∗0.05 > *p* < 0.0001∗∗). *D*, HEK cells transfected with Cry2–DAT were treated with cytochalasin D to inhibit actin polymerization. The cells were then with treated with the vehicle, 10 μM METH, 10 μM NOM, or *blue light* as indicated (n = 5–9, *p* < 0.0001). *E*, IDT307 uptake shows that 488-nm pulse before 10 μM METH or 200 nM PMA treatment does not inhibit downregulation of multimerized Cry2–DAT. IDT307 uptake is reduced when 488-nm pulse is followed with 10 μM METH or 200 nM PMA treatment (n = 15, *p* ≤ 0.0001). Cry2, cryptochrome 2; Cry2–DAT, cryptochrome 2 and DAT with an mCherry fluorescent reporter; DAT, dopamine transporter; METH, methamphetamine; NOM, nomifensine; PMA, phorbol 12-mristate 13-acetate.

Because our data demonstrating that cytochalasin D (CD) treatment inhibited increased MFZ 9-18 binding and IDT307 uptake at the PM in Cry2–DAT–expressing cells upon the blue light–stimulated formation of multimers, we exploited live-cell, confocal time lapse experiments, to assess the redistribution of Cry2–DAT in light-stimulated cells. Figure 5*A* provides representative snapshots of confocal images before and after blue light stimulation and demonstrate that exposure to blue light increases Cry2–DAT (red fluorescence) at the PM (thick yellow arrows) and a concomitant decrease of Cry2–DAT intracellularly (thin white arrows).

Applying the same experimental treatment conditions used for IDT307 uptake and MFZ 9-18 binding experiments, we performed cell surface biotinylation on Cry2–DAT–expressing cells to confirm if the increased DAT uptake and binding correlated with increased accumulation of DAT at the membrane. METH treatment resulted in a significant decrease in the cell surface DAT (*p* < 0.05), and significantly more Cry2–DAT (*p* < 0.001) was detected at the PM after blue light stimulation (Figs. 5, *B* and *C*, and S3*D*). Blue light stimulation of Cry2–DAT multimers, under control conditions, showed an increase in DATs at the PM, but the increase was not significant (*p* < 0.40). NOM treatment resulted in a significant decrease in cell surface expression, but no change in cell surface DATs was detected after blue light stimulation (Figs. 5, *B* and *C*, and S3*D*). Cross-linking before biotinylation did not definitively resolve DAT multimeric pattern at the PM beyond the dimer, although we detected a robust increase of Cry2–DAT at the PM ranging from monomer to multimeric forms with NOM after blue light multimerization (Fig. S3, *A–C*). These higher order multimers could be minimal or transient in nature at the PM or associated with other proteins causing the indistinct smear observed. In addition, cross-linking has been shown to effectively capture DAT multimers after COC treatment (12, 15). Because NOM and COC block DAT reuptake of DA, the increased surface protein detection with cross-linking and subsequent biotinylation with NOM may capture the increased cell surface Cry2–DAT not previously detected with biotinylation alone (Fig. S3, *A–C*).

To further explore the possibility that blue light multimerization of Cry2–DAT impacts its trafficking, we treated cells with CD to inhibit bulk trafficking of Cry2–DAT to and from the PM before examining Cry2–DAT MFZ 9-18 binding. Pretreatment with CD inhibited the increased binding of MFZ 9-18 that resulted from blue light–induced Cry2–DAT multimerization in control, METH and NOM treatments (Fig. 5*D*). Taken together, these data suggest that the increase in uptake and binding after blue light–induced Cry2–DAT multimerization results in increased trafficking of Cry2–DAT to the cell surface.

Next, we tested if blue light–induced Cry2–DAT multimerization stabilized Cry2–DAT at the PM. In contrast to the previous experiments, the blue light pulse was performed first and followed by treatment with 10 μM METH or 200 nM phorbol 12-mristate 13-acetate (PMA) for 2 min, and subsequently, the uptake was measured (Fig. 5*E*). Multimerization did not rescue the downregulation of Cry2–DAT by METH or PMA when blue light stimulation was performed before METH or PMA treatment. This suggests that multimerization does not stabilize DAT at the PM.

### Blue light–multimerized Cry2–DAT colocalizes with Rab11

**Figure 6:**
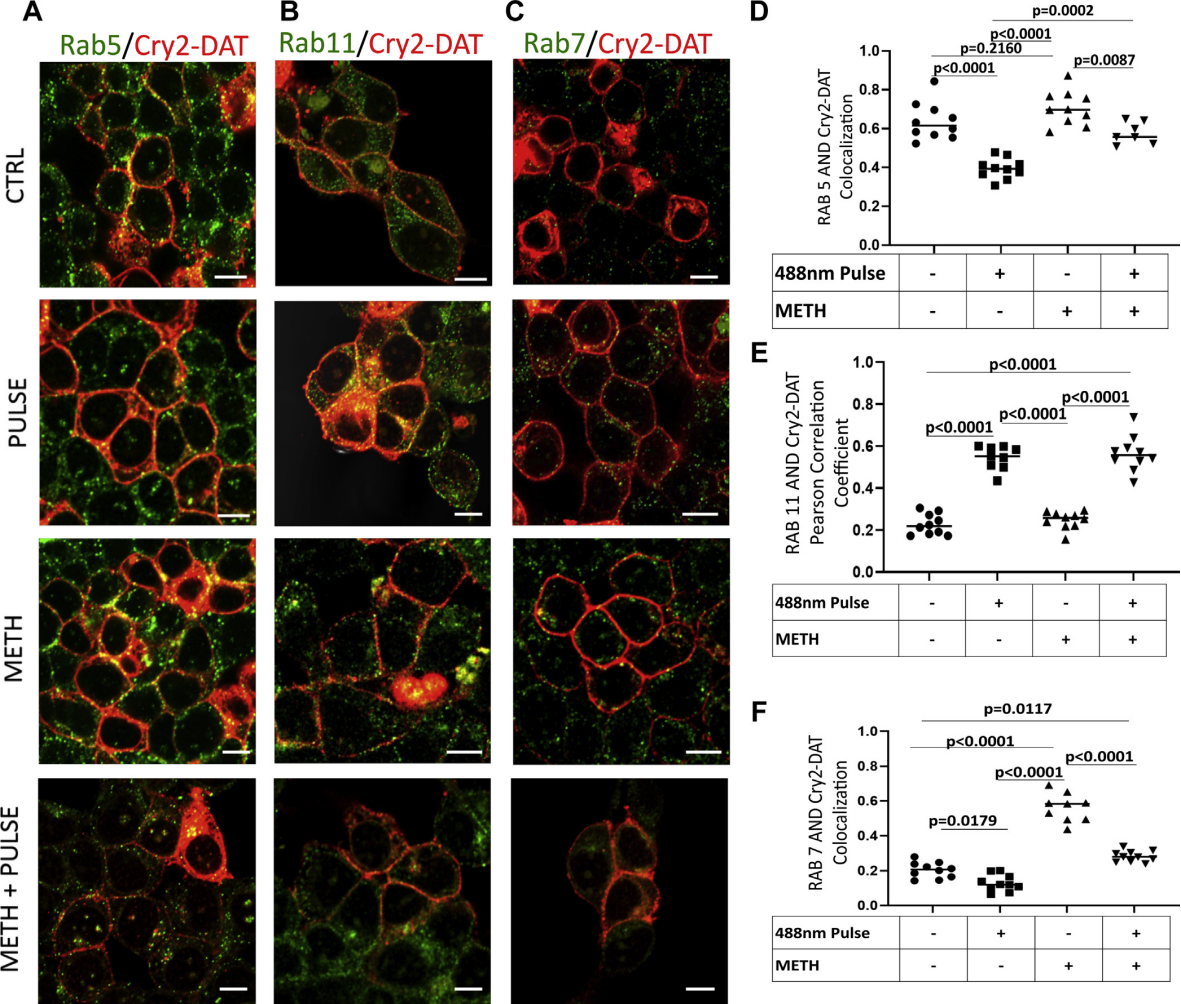
***Blue light*–multimerized Cry2–DAT colocalizes with Rab11.** HEK cells were transfected with Cry2–DAT and treated with vehicle or 10 μM METH and exposed with 488-nm light where indicated. *A*, Cry2–DAT (*red*) and Rab5 (*green*), (*B*) Rab11 (*green*), and (*C*) Rab7 (*green*) was used to test colocalization (*yellow*) with 10 μM METH treatment and a 488-nm light pulse. *D*, Pearson’s correlation coefficient was used to determine the extent of colocalization of Cry2–DAT with Rab5, (*E*) Rab11, and (*F*) Rab7 (n = 7–10). The scale bar represents 10 μm. Cry2–DAT, cryptochrome 2 and DAT with an mCherry fluorescent reporter; METH, methamphetamine.

We next examined a series of Rab proteins to assess the trafficking pathways utilized by multimerized Cry2–DAT, leading to increased cell surface expression of blue light–stimulated Cry2–DAT. FLAG–DAT and Cry2–DAT colocalization with Rab4, 5, 7, and 11 were assessed using Pearson's correlation coefficient for green (Rab) and red (Cry2–DAT or FLAG–DAT) immunostained cells. None of the experimental conditions induced changes in colocalization with Rab4 and Cry2–DAT or FLAG–DAT (Pearson’s correlation coefficient of ≤ 0.30) (data not shown), one of two recycling endosomal proteins previously identified for DAT recycling to the cell surface (45). In FLAG–DAT–expressing cells, no significant changes in colocalization of FLAG–DAT and Rab5 were detected (Table 1). 10 μM METH treatment resulted in significantly increased colocalization of FLAG–DAT with Rab11 (Pearson’s correlation coefficient of 0.415 ± 0.016) and Rab7 (Pearson’s correlation coefficient of 0.580 ± 0.030). Blue light stimulation did not alter the colocalization of FLAG–DAT with Rab5, 7, or 11 (Table 1). In control conditions, Cry2–DAT and Rab5 colocalization was strong (Pearson’s correlation coefficient of 0.638 ± 0.030), indicative of constitutive trafficking from the PM. In contrast, Cry2–DAT had very little association with the other Rab proteins tested in control conditions (Fig. 6 and Table 2). Blue light stimulation to multimerize Cry2–DAT resulted in decreased Cry2–DAT/Rab5 association (Pearson’s correlation coefficient of 0.394 ± 0.017) but significantly increased colocalization with Rab11 recycling compartments (Pearson’s correlation coefficient of 0.546 ± 0.019). 10 μM METH treatment increased Rab5 (Pearson’s correlation coefficient of 0.706 ± 0.028) and Rab7 (0.509 ± 0.059) colocalization with Cry2–DAT. 10 μM METH pretreatment followed by multimerization of Cry2–DAT resulted in a significant decrease in colocalization with Rab7 (Pearson’s correlation coefficient of 0.283 ± 0.010) and a significant increase in colocalization with Rab11 (Pearson’s correlation coefficient of 0.563 ± 0.027) compared with METH treatment alone (Fig. 6 and Table 2). These data indicate that multimerized Cry2–DAT primarily associates with Rab5- and Rab11-positive endosome but not Rab4- or Rab7-positive endosomal compartments.

**Table 1:** FLAG–DAT colocalization with endosomal Rab proteins in HEK293 cells

Rab protein	Control	Pulse	METH	METH/pulse
Rab5	0.634 ± 0.005	0.639 ± 0.007	0.671 ± 0.003	0.665 ± 0.007
Rab11	0.223 ± 0.022	0.231 ± 0.013	0.415 ± 0.016	0.415 ± 0.016
Rab7	0.234 ± 0.009	0.221 ± 0.029	0.580 ± 0.030	0.523 ± 0.019

Data are the mean Pearson’s correlation coefficient ± SE.

**Table 2:** Cry2–DAT colocalization with endosomal Rab proteins in HEK293 cells.

Rab protein	Control	Pulse	METH	METH/pulse
Rab5	0.638 ± 0.030	0.394 ± 0.017	0.706 ± 0.028	0.576 ± 0.021
Rab11	0.227 ± 0.016	0.546 ± 0.019	0.248 ± 0.013	0.563 ± 0.027
Rab7	0.204 ± 0.014	0.129 ± 0.015	0.509 ± 0.059	0.283 ± 0.010

Data are the mean Pearson’s correlation coefficient ± SE.

### Blue light stimulation induces exocytic trafficking

**Figure 7:**
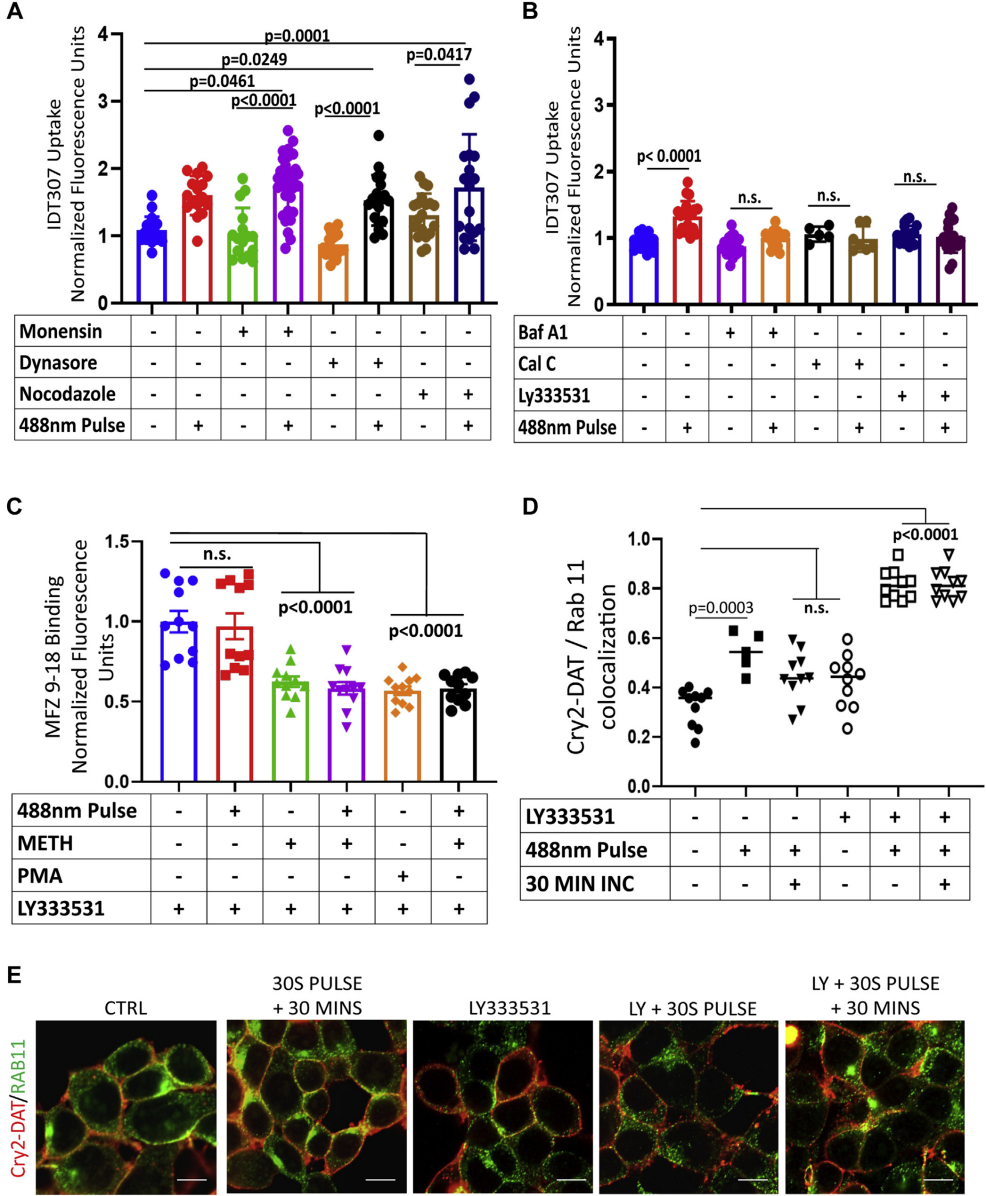
***Blue light* stimulation induces exocytic trafficking.***A*, HEK cells were transfected with Cry2–DAT, and IDT307 uptake was measured in cells pretreated with monensin (2.5 μM), dynasore (80 μM), and nocodazole (2 μM) before 488-nM *blue light* pulse. (n = 12–28, 0.05 > *p* < 0.01). *B*, HEK cells were transfected with Cry2–DAT, and IDT307 uptake was measured after pretreatment with calphostin C (1 μM), bafilomycin A1 (0.06 nM), and LY333531 (0.6 nM) and exposed to a 488-nm light pulse where indicated. (n = 11–28, 0.05 > *p* < 0.01). *C*, HEK cells were transfected with Cry2–DAT, and MFZ 9-18 binding was measured after pretreatment with METH, and PMA followed by LY333531 (0.6 nM), and exposed to a 488-nm light pulse where indicated (n = 12–28, 0.05 > *p* < 0.01). *D*, HEK cells were transfected with Cry2–DAT and treated with the vehicle or 10 μM METH and exposed with 488-nm light where indicated. Pearson’s correlation coefficient was used to determine the extent of colocalization of Cry2–DAT with Rab11 with pretreatment of LY333531 (0.6 nM), exposed to 488-nm light for 30 s, or allowed to incubate for 30 min after treatment as indicated (n = 6–10). *E*, representative images of Cry2–DAT (*red*) and Rab11 (*green*) was used to test colocalization (*yellow*) with pretreatment of LY333531 (0.6 nM), exposed to 488-nm light for 30 s, or allowed to incubate for 30 min after treatment as indicated. The scale bar represents 10 μm. Cry2–DAT, cryptochrome 2 and DAT with an mCherry fluorescent reporter; METH, methamphetamine; n.s., not significant; PMA, phorbol 12-mristate 13-acetate.

To identify the trafficking itinerary of multimerized Cry2–DAT in greater detail, we examined the impact of several endosomal trafficking inhibitors on Cry2–DAT multimer–induced increase in IDT307 uptake activity. Treatment of Cry2–DAT cells with monensin (targeting early and late endosomal maturation steps) (45, 46), dynasore (internalization and early endosomal maturation) (47) and nocodazole (targeting late endosomal maturation) (48) had no impact on multimer-induced increase in uptake activity (Fig. 7*A*). Further studies on membrane binding with MFZ 9-18 demonstrated that multimerization using blue light stimulation recovered the membrane binding in cells pretreated with monensin or nocodazole alone and with subsequent treatment with 10 μM METH (Fig. S4, *A* and *B*). These data demonstrate that early and late endosomal compartments (Rab 5 and 7, respectively) are not involved in mobilization of the DAT to the PM upon multimerization (Fig. 7*A*)

**Figure 8:**
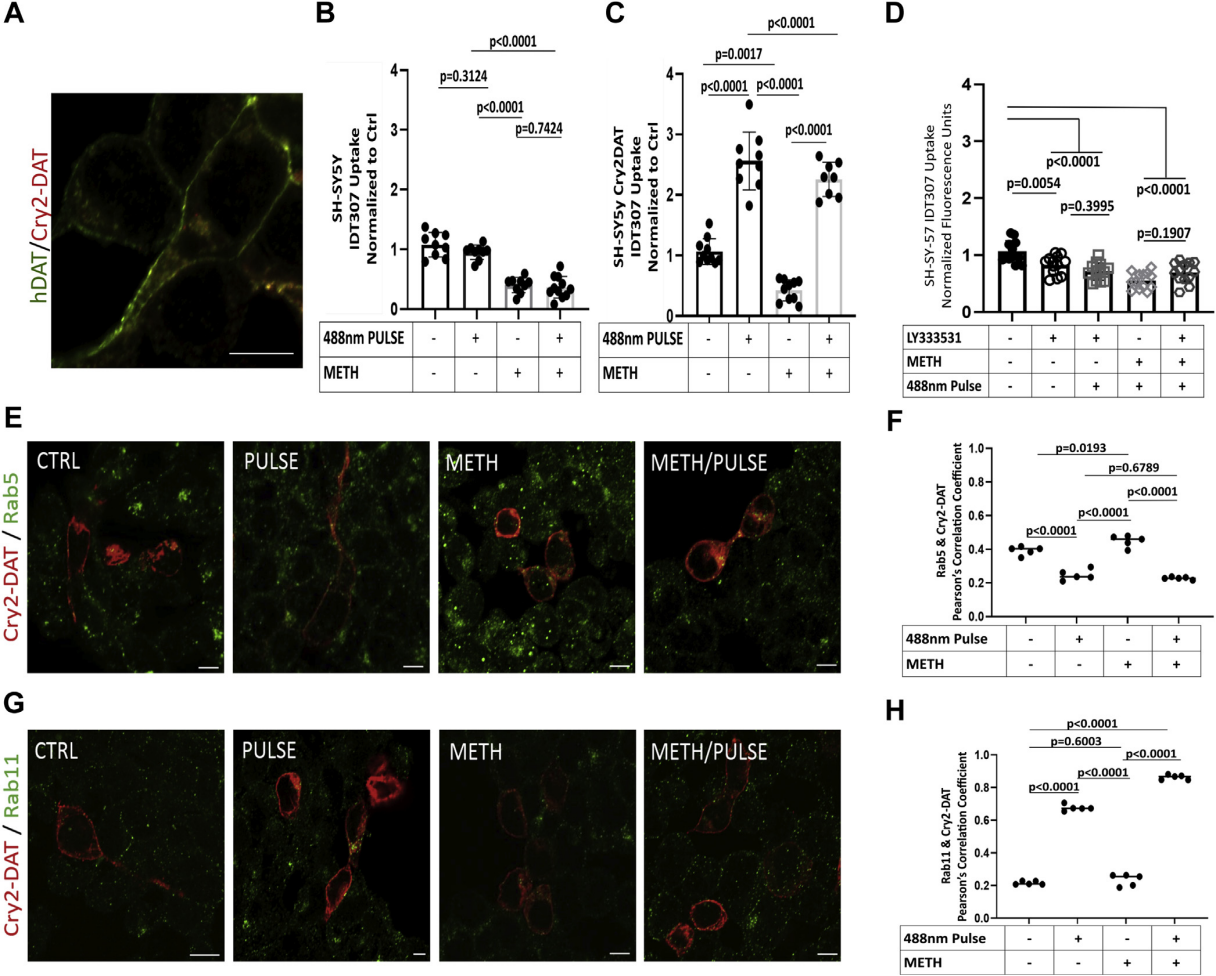
**Novel Cry2–DAT–multimerized uptake and trafficking mechanisms are confirmed in SHSY-5Y differentiated cells.***A*, immunofluorescence staining of differentiated SH-SY5Y cells show the endogenous DAT (*green*) and Cry2–DAT (*red*) colocalization. *B*, uptake of IDT307 was measured in differentiated SH-SY5Y cells treated with vehicle or 10 μM METH and exposed to a 488-nm light pulse where indicated (n = 9–10, 0.01> *p* < 0.001). *C*, uptake of IDT307 was measured in differentiated SH-SY5Y cells expressing Cry2–DAT and treated with vehicle or 10 μM METH and exposed to a 488-nm light pulse where indicated (n = 9–10, 0.01 > *p* < 0.001). *D*, uptake of IDT307 was measured in differentiated SH-SY5Y cells expressing Cry2–DAT and treated with vehicle or 10 μM METH followed by LY333531 exposed to a 488-nm light pulse where indicated (n = 9–10, 0.01 > *p* < 0.001). *E*, differentiated SH-SY5Y cells were transfected with Cry2–DAT (*red*) and imaged for colocalization with Rab5 (*green*) and (*G*) Rab11 (*green*). *F*, Pearson’s correlation coefficient was used to determine the extent of colocalization of Cry2–DAT with Rab5 and (*H*) Rab11 (n = 5, *p* < 0.0001). The scale bar represents 10 μm. Cry2–DAT, cryptochrome 2 and DAT with an mCherry fluorescent reporter; METH, methamphetamine.

**Figure 9:**
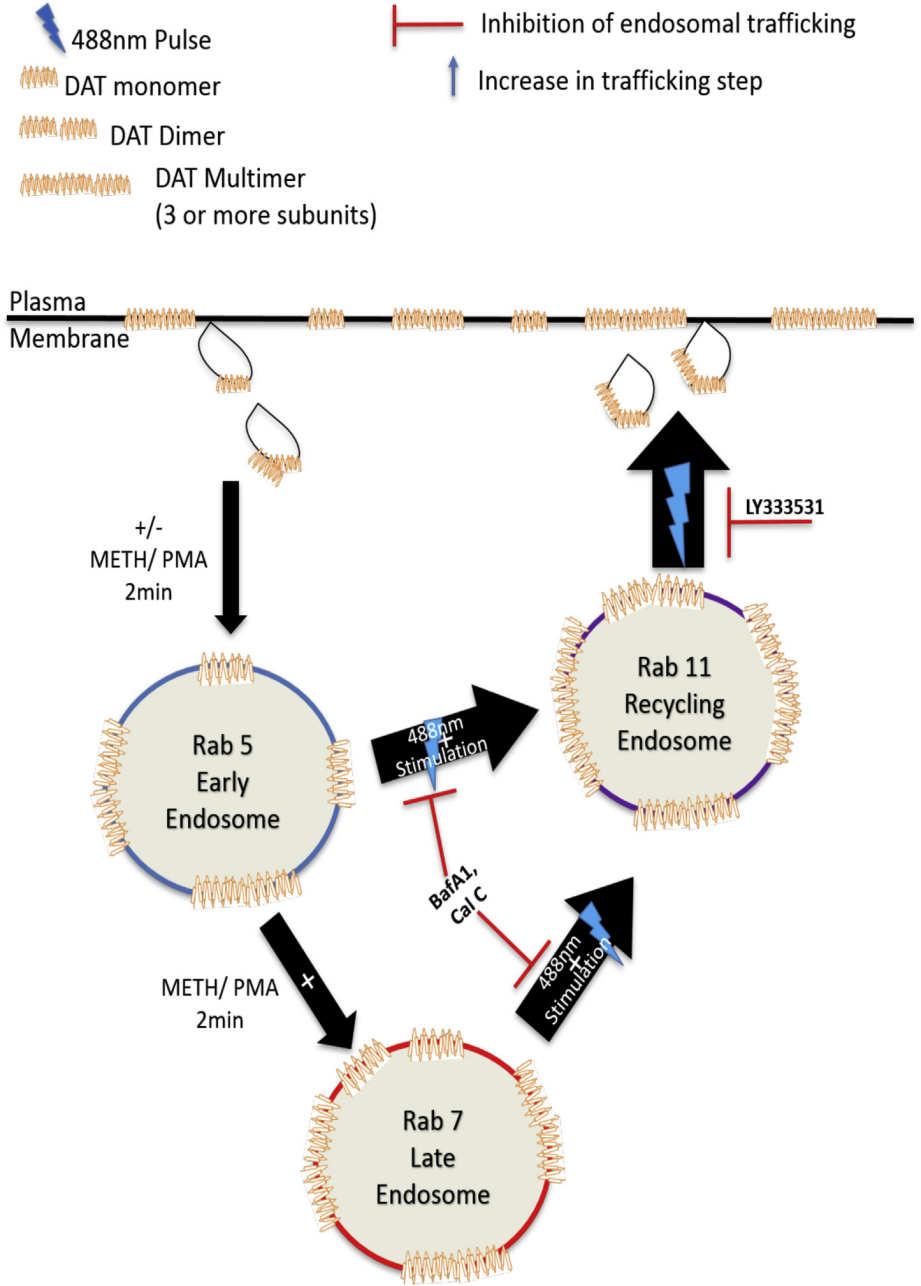
**Proposed mechanism for Cry2–DAT multimer–induced increase in uptake and binding.** Cry2–DAT constitutively traffics to Rab5 early endosomes, Rab11 recycling endosomes, and back to the plasma membrane. Treatment with 10 μM METH or 200 nM PMA induces Cry2–DAT downregulation from the plasma membrane to traffic to Rab5- and Rab7-associated endosomes. Light stimulation (488 nm) of Cry2–DAT mobilizes Cry2–DAT to traffic from Rab11 endosomes to the plasma membrane to increase Cry2–DAT uptake and binding. This can be impeded by inhibiting the maturation of recycling endosomes (using Cal C or Baf A1) or inhibiting PKCβ. Baf A1, bafilomycin A1; Cal C, calphostin C; Cry2–DAT, cryptochrome 2 and DAT with an mCherry fluorescent reporter; METH, methamphetamine; PMA, phorbol 12-mristate 13-acetate.

In the same manner, we tested recycling inhibitors calphostin C (Cal C), bafilomycin A1 (Baf A1) and LY333531 (LY). Cal C, which targets the maturing endosomal compartments and recycling endosomal maturation in a PKC-dependent manner (49), significantly increased the uptake compared with the control; however, subsequent blue light stimulation did not enhance IDT307 uptake activity in the presence of Cal C (Fig. 7*B*). Baf A1 and LY were used to confirm these findings. Baf A1 inhibits the maturation of recycling endosomes in a pH-dependent manner (48). LY is a selective PKCβ inhibitor. PKCβ has been implicated as the mechanism that contributes to regulating DAT PM expression (50). Multimer-induced increase in IDT307 uptake was blunted with Baf A1 and LY pretreatment, similar to Cal C treatment (Fig. 7*B*). These data are consistent with the interpretation that endosomal recycling compartments serve as the pathway that is used by blue light–activated multimers to achieve an accelerated arrival to the cell surface (Fig. 9).

To further test this interpretation, MFZ 9-18 binding assay was performed using the PKCβ inhibitor LY. LY treatment did not allow the recovery of membrane binding upon subsequent blue light stimulation. LY treatment followed by 10 μM METH or 200 nM PMA treatment had a decrease in MFZ 9-18 binding; blue light stimulation also did not recover MFZ 9-18 binding in Cry2–DAT–expressing HEK293 cells. These data support the IDT307 uptake data and Rab staining data above. In particular, PKCβ inhibition reveals the mechanism that is used to mobilize multimer-induced increase in trafficking and membrane binding.

Finally, to confirm PKCβ as the mechanism for mobilizing the multimer-induced trafficking to the membrane, we performed staining on Cry2–DAT cells to observe changes in colocalization with Rab11 in the presence and absence of LY. Blue light exposure on Cry2–DAT–expressing cells was shown to increase Rab11 colocalization. If cells stimulated with blue light were incubated in the dark for 30 min after the light pulse, the colocalization decreases, demonstrating the movement of Cry2–DAT into and out of Rab11 pools upon pulsing (Fig. 7, *D* and *E*). LY treatment, however, trapped multimerized DAT in Rab11 endocytic compartments, restricting its movement out of Rab11 compartments, which is demonstrated by the increase in Cry2–DAT/Rab11 colocalization in cells treated with LY and then pulsed for 30 s and incubated for 30 min (Fig. 7, *D* and *E*). These data suggest that blue light–stimulated Cry2–DAT multimerization favors an exocytic trafficking mechanism from Rab11-positive endosomes to increase uptake and binding (Fig. 9).

### Novel Cry2–DAT–multimerized uptake and trafficking mechanisms confirmed in differentiated human dopaminergic neuronal-like cells

To establish that our findings with multimerized Cry2–DAT were consistent in dopaminergic cells, we examined Cry2–DAT expression, uptake activity, and trafficking in differentiated human dopaminergic neuronal-like SH-SY5Y cells. Cry2–DAT (red) and endogenously expressed DAT (green) had a Pearson’s correlation coefficient of 0.85, indicating a high-degree colocalization (Fig. 8*A*). Consistent with our uptake measurements in FLAG–DAT–expressing HEK cells, the uptake of IDT307 in differentiated SH-SY5Y cells was significantly decreased with METH pretreatment, and blue light stimulation did not affect uptake (Fig. 8*B*). However, when Cry2–DAT was expressed in differentiated SH-SY5Y cells, blue light stimulation to multimerize Cry2–DAT significantly increased uptake by 2.5-fold compared with the control cells not exposed to blue light stimulation. METH pretreatment significantly decreased the uptake in Cry2–DAT–expressing differentiated SH-SY5Y cells, and blue light multimerization resulted in a robust and significant increase in uptake after METH pretreatment similar to control with blue light stimulation (Fig. 8*C*). These data demonstrate that changes in uptake in Cry2–DAT–expressing differentiated SH-SY5Y cells are similar to those observed in Cry2–DAT–expressing HEK cells.

The role of trafficking pathways on Cry2–DAT expression in differentiated SHSY-5Y cells was analogous to previous findings in HEK293 cells.

The effect of LY on multimer-induced increase in IDT307 uptake was tested in Cry2–DAT–expressing differentiated DA SHSY-5Y cells. LY inhibited the recovery of IDT307 uptake upon blue light stimulation. Blue light stimulation to multimerize Cry2–DAT resulted in significantly decreased colocalization with Rab5 (Pearson’s correlation coefficient of 0.2478 ± 0.018) and METH-pretreated samples (Pearson’s correlation coefficient of 0.228 ± 0.003) and significantly increased colocalization with Rab11 under control (Pearson’s correlation coefficient of 0.658 ± 0.017) and in METH-pretreated conditions (Pearson’s correlation coefficient of 0.874 ± 0.004) (Fig. 7, *D*–*G* and Table 3), suggesting that blue light–stimulated Cry2–DAT multimerization favors a recycling trafficking mechanism to populate DAT at the cell surface (Fig. 9).

**Table 3:** Cry2–DAT colocalization with endosomal Rab proteins in differentiated SH-SY5Y cells

Rab protein	Control	Pulse	METH	METH/pulse
Rab5	0.392 ± 0.012	0.248 ± 0.014	0.449 ± 0.016	0.228 ± 0.003
Rab11	0.242 ± 0.026	0.658 ± 0.017	0.242 ± 0.024	0.874 ± 0.004

Data are mean Pearson’s correlation coefficient ± SE.

## Discussion

The optogenetic Cry2–DAT model presented herein represents a new approach to understanding the importance of how DAT multimerization affects its localization and function in the absence and simultaneous presence of DAT-binding ligands. Our data revealed that multimerized Cry2–DAT increased IDT307 and uptake capacity by inducing rapid delivery of naïve DAT to the PM and by upregulating trafficking to Rab11-recycling endosomes. To our knowledge, this is the first report of a ligand-independent characterization of DAT multimer function using optogenetics.

Prior studies showed evidence for the formation of DAT multimers upon treatment with METH, COC, or AIM-100 (8, 12, 51). METH treatment competitively inhibits DA uptake and induces DAT downregulation at the PM (52, 53, 54, 55, 56), and the data in Figure 2, *A* and *B* show for the first time that DA induces YFP–DAT multimers in a rapid time-dependent manner distinct from METH-induced multimers. COC blocks DA uptake, but prior studies have not linked COC-induced multimers with COC-induced increases in DAT membrane expression (14, 40). The binding of AIM-100 to the DAT promotes the formation of DAT multimers and induces endocytosis, decreasing DAT uptake, although there is not a clear consensus of the functional mechanisms of how AIM-100 acts on the DAT (51, 57). Loss-of-function mutation studies show that the formation of multimers with the WT and mutant DAT results in a decrease in DAT function at the PM (8).

Our novel, optogenetic model allows us to control DAT multimer formation with high spatiotemporal precision in absence of binding ligands or cross-linkers or introducing amino acid mutations in the DAT. This model relies on the blue light sensitivity of the Cry2 portion, N-terminal to the mCh fluorescent reporter (Fig. 1*A*), of the Cry2–DAT construct to form higher order multimers of the DAT. The multimerization of Cry2 is very robust and may lead to Cry2–DAT multimers with no functional relevance or to more DAT multimers than would be generated under physiological conditions. Although this method of DAT multimerization may not completely recapitulate the exact mechanism of DAT multimerization, our results point to many similar characteristics as YFP–DAT.

The expression of Cry2–DAT was similar to that of YFP–DAT in HEK cells and colocalized with the native DAT when expressed in differentiated SH-SY5Y cells (Figs. 1*C* and 7*A*). Basal IDT307 uptake of Cry2–DAT was comparable with that of FLAG–DAT in HEK cells and differentiated DAT-expressing SH-SY5Y cells (Figs. 1*D* and 7). We found that Cry2–DAT, like YFP–DAT, is a dimer at the PM and blue light multimerization of Cry2–DAT formed higher order multimers comparable with DA and METH in YFP–DAT–expressing cells (Figs. 2 and S3).

Our FRAP and FRET analyses revealed that YFP–DAT could be incorporated in Cry2–DAT multimers after blue light stimulation. Cry2–DAT has similar diffusion characteristics as YFP–DAT before multimerization, but after multimerization, Cry2–DAT diffusion is significantly slower, as is expected for larger molecular complexes. Pretreatment with METH and subsequent blue light multimerization of Cry2–DAT significantly reduced D compared with either condition alone (Fig. 3*C*). It is tempting to speculate that the lowered D from METH treatment could be due to the higher order multimers of the DAT previously reported (52), although several other factors could affect the diffusion of the DAT in the presence of METH such as interacting proteins and membrane nanodomains (58). In cells coexpressing YFP–DAT and Cry2–DAT, the diffusion of YFP–DAT was slower with blue light induction of Cry2–DAT multimers, but the M_f_ was increased. The M_f_ is an indication of the fraction of labeled proteins capable of recovering into the photobleached area. Increases in M_f_ indicate fewer barriers to diffusion or an increase in protein delivered to the photobleached region of the cell outside of the x-y diffusing plane, for example, from the cytoplasm to the PM (59). However, fewer diffusional barriers primarily coincide with an increase in D (60). Because we measured a significantly lower diffusion rate, these data suggest that the new DAT is trafficking to the cell surface—in what we propose is accelerated delivery of the multimerized DAT to the PM. Although our FRAP data cannot definitively distinguish if DAT is trafficking to the PM with blue light multimerization, given our additional supporting data, it is likely the M_f_ in context of D may be an indication of newly trafficked transporter to the cell surface.

Although FRAP suggested that YFP–DAT and Cry2–DAT were diffusing together, FRET analysis confirmed that YFP–DAT and Cry2–DAT were in close proximity, forming YFP–DAT and Cry2–DAT multimeric complexes. METH treatment alone and blue light stimulation alone to multimerize Cry2–DAT showed a similar FRET efficiency between YFP–DAT and Cry2–DAT, validating the optogenetic approach as representative of DAT multimerization. Taken together, the FRAP and FRET results reveal that YFP–DAT was incorporated into blue light–stimulated Cry2–DAT multimers and that this optogenetic approach represents the assembly of the DAT into multimers. The blue light–sensitive Cry2 portion of the Cry2–mCh–DAT construct is a means to drive multimer formation in the absence of substrates, inhibitors, or allosteric modulators of the DAT, allowing us to directly test the function of DAT multimers.

Based on previous finding with METH induction of DAT multimers, we expected to see a decrease in uptake and binding capacity of blue light–induced Cry2–DAT multimers. Surprisingly, we found that inducing Cry2–DAT multimers with blue light significantly increased uptake capacity (Fig. 4, *B* and *D*). We found that pretreatment with 10 μM METH significantly reduced Cry2–DAT uptake, as expected. However, when Cry2–DAT was multimerized with blue light after pretreatment with METH, uptake significantly increased (Fig. 4, *B* and *D*). This finding for Cry2–DAT in HEK293 cells was corroborated in differentiated human dopaminergic neuronal-like SH-SY5Y cells, where the increase in the uptake after multimerization in SH-SY5Y cells was even more pronounced (Fig. 8*C*). Similarly, 10 μM NOM significantly decreased Cry2–DAT uptake, and multimerization of Cry2–DAT significantly increased the uptake after NOM pretreatment (Fig. 4, *B* and *D*).

To determine the availability of Cry2–DAT–binding sites after blue light–induced multimerization, we used MFZ 9-18, a fluorescent COC analogue. Pretreatment of Cry2–DAT with METH or NOM decreased the binding of MFZ 9-18 as expected, but blue light multimerization significantly increased MFZ 9-18 binding (Fig. 4*F*), analogous to the increased transport activity observed in IDT307 uptake experiments. Multimerized Cry2–DAT had significant increases in MFZ 9-18 binding with both pretreatments, suggesting that there is an increase in the outward-facing transporter available at the cell surface.

Because the increased uptake and binding capacity of Cry2–DAT after multimerization could be due to a conformational change, increased trafficking to the cell surface, or a combination of a conformational change and trafficking, we examined the role of trafficking in more detail. It is well known that METH induces trafficking of the DAT away from the cell surface and undergoes a conformational change from outward- to inward-facing conformation (58, 61, 62), whereas NOM stabilizes the cell surface DAT in the outward-facing conformation similar to COC but with slightly higher binding affinity to the DAT (63). Because NOM stably binds to the DAT in the outward-facing conformation and METH induces an inward-facing conformation, the increased uptake and binding data for NOM and METH pretreatment followed by multimerization of Cry2–DAT suggest naïve DAT trafficking to the PM to bind MFZ and IDT307. To confirm this, cells were pretreated with cytochalasin D, an actin polymerization inhibitor, to halt the trafficking (47). If the increase in transport with multimerization is due to a conformational change, rather than trafficking, light-induced increases in uptake and binding would be detected in the presence of cytochalasin D. However, no changes in uptake or binding were detected with blue light–induced multimerization of Cry2–DAT in cells pretreated with cytochalasin D (Fig. 5*D*), suggesting a role for trafficking in the increased transport activity that parallels multimerization. Cell surface biotinylation and Western blot analysis indeed revealed that more DAT is detected at the cell surface with METH pretreatment followed by blue light multimerization of Cry2–DAT than METH treatment alone (Figs. 5, *B* and *C* and S3*D*). When we used crosslinking followed by biotinylation, we were able to detect an increase in cell surface Cry2–DAT after NOM treatment and blue light pulse (Fig. S3, *A–C*). With the two methods, we were able to detect increased cell surface Cry2–DAT with METH followed by blue light pulse with biotinylation and NOM followed by blue light pulse with crosslinking followed by biotinylation; however, we were unable to detect these changes with only one method. This could be due to molecular changes in DAT structure with METH and NOM, which allows one method to work better for each compound.

Live-cell imaging of Cry2–DAT after inducing multimers with blue light confirmed the increase in fluorescent intensity of Cry2–DAT at the surface with concomitant reduction in the cytoplasmic signal (Fig. 5*A*), suggesting that the significant increase in uptake and binding of multimerized Cry2–DAT is due to rapid trafficking (<6 min) of Cry2–DAT to the PM from internal pools of the DAT. Indeed, several prior DAT-trafficking studies support these findings. It has been shown that the DAT must be at least in dimers to exit the endoplasmic reticulum and traffic to the PM (8, 9, 12). Although AMPH results in a loss of the DAT at the PM after 2 min, within 30 s to 2 min, a rapid upregulation in cell surface DAT expression was detected in rat striatal synaptosomes and DAT-expressing HEK293 cells (64). These same investigators showed that DA, in addition to AMPH, also elicited an increase in DAT at the PM within the same time frame (50), and we found that within 2 min, DA and METH induce DAT multimers at the PM (Fig. 2, *A* and *B*).

The redistribution of proteins at the PM contributes to synaptic plasticity. For the DAT, its PM redistribution has a role in maintaining presynaptic plasticity and DA homeostasis (65). An increased number of DATs at the cell surface is particularly important in response to DAT downregulation or blocked activity (14, 15, 64, 65). Our findings indicate that multimerization induces redistribution of Cry2–DAT to the PM *via* a rapid trafficking mechanism. Our immunofluorescence data revealed that multimerized Cry2–DAT decreases colocalization with Rab5 and Rab7 endosomes and increases colocalization with Rab11 in HEK293 and SHSY-5Y cells (Figure 6, Figure 7, Figure 8), suggesting that multimerization could alter DAT’s trafficking itinerary from early and late endosome to Rab11 recycling endosomes to facilitate the delivery of multimerized DAT to the cell surface. PKCβ has been reported to be responsible for the delivery of the DAT to the cell surface from Rab11 compartments within 30 s to 2 min after AMPH treatment (42, 66). Using an LY as a specific inhibitor of PKCβ, the increase in IDT307 uptake and MFZ 9-18 binding was inhibited. Immunofluorescence staining demonstrates changes in Cry2–DAT Rab11-associated pools upon and after multimerization in a time-dependent manner (Figs. 6, *B* and *E*, and 7*E*). These results are also consistent the finding that the rapid upregulation of the DAT at the PM after AMPH treatment was found to be regulated by PKCβ and Rab11-positive endosomes (66, 67).

Recent studies demonstrated that with constitutive DAT trafficking, there is colocalization of the DAT with Rab11, and this colocalization is significantly increased after DAT-stimulated endocytosis (68). It has been proposed that constitutive trafficking of the DAT allows for a quick and efficient mechanism to maintain DA homeostasis (69). It is possible that DAT multimerization is a key component of this mechanism to fine-tune DA homeostasis, and this could explain why the multimeric DAT has been detected after treatment with DA, COC, and METH by us (Fig. 2, *A* and *B*) and others (9, 15, 52).

The DAT plays a vital role in normal DA neurotransmission, and because it is a target for addictive drugs and involved in dopaminergic diseases, the cellular mechanisms controlling DAT availability at the PM can greatly influence behavior and associated pathologies. In this study, we developed an optogenetic DAT model to investigate with spatiotemporal precision, how DAT multimerization may contribute to DA homeostasis. Our data show that inducing DAT multimers significantly increases DAT uptake and binding in the vehicle, METH, and NOM pretreatment conditions. The increase in DAT function results from a rapid mobilization to the cell surface. The increased colocalization of Cry2–DAT after multimerization with Rab11 and decreased presence in Rab5 and Rab7 endosomes suggest that the multimeric DAT may prime trafficking pathways for delivery to the cell surface to maintain tight control of extracellular DA (Fig. 9). However, in absence of optogenetic controls, it remains to be determined what cellular components control DAT multimerization. Perhaps future investigations into the mechanisms controlling DAT multimerization could be developed for therapeutic interventions to fine-tune DA levels in psychiatric disorders.

## Experimental procedures

### Materials and reagents

The materials and reagents used for this study can be found in Table 4. The antibodies used for this study experiments can be found in Table 5.

**Table 4:** Materials used to complete experiments complemented with catalog numbers, vendors, and concentrations

Chemical	Vendor name	Catalog number	Working concentration	Experiment
IDT307	Sigma-Aldrich	SML0756	10 μM	IDT uptake
NP-40 Buffer	Life Technologies	FNN0021		Surface biotinylation
Sodium deoxycholate	Thermo Fisher	89904		NP-40 buffer
Copper sulfate	Sigma-Aldrich	61230-100G	100 μM	Multimer determination assay
1,10-Phenanthroline	Sigma-Aldrich	131377-5G	400 μM	Multimer determination assay
N-Ethylmaleimide (NEM)	Sigma-Aldrich	E1271-5G	10 μM	Multimer determination assay
Retinoic acid	Sigma-Aldrich	R2625		
Glycine	Sigma-Aldrich	G8898	1.25 μM	
Formaldehyde	Sigma-Aldrich	F8775	1%	
Membrane Perm kit	Thermo Fisher	89842		Multimer determination assay
Methamphetamine	Sigma Aldrich	M8750	10 μM	
Dopamine hydrochloride	Abcam	ab120565	10 μM	Multiple experiments
Nomifensine	Sigma-Aldrich	N1530	10 μM	IDT uptake
MatTek dishes	MatTek Corporation	P35G-1.5-14-C		Microscopy experiments
35-mm dishes	CELLSTAR	627160		
100-mm dishes	Thermo Scientific	172931		
60-mm dishes	CELLSTAR	628160		
Glass coverslips	Thermo Fisher	18CIR-1.5		
Qiagen Midi Prep kit	Qiagen	12143		
NeutrAvidin agarose resin	Thermo Fisher	29201	100 μL	Surface biotinylation
Sulfo-NHS-S-S-biotin	Thermo Fisher	21331	2 μM	Surface biotinylation
DMEM	Gibco	11965	1 bottle	Cell culture
Penicillin/Streptomycin	Gibco	15140	10,000 units/mL	Cell culture
Lipofectamine	Invitrogen	11668	1:3 dilution	Cell culture
FBS (heat inactivated)	Atlanta Biologicals	S11150H	10%	Cell culture media
HEK293 cells	ATCC	CRL-1573		Cell culture, Cry2–DAT transient transfection
Pierce Protease Inhibitor Tablets	Thermo	A32963	1×	Multimer determination assay
Diamond Antifade Mounting Media with DAPI	Invitrogen	P36962		Staining
Immun-Blot PVDF membrane	Bio-Rad	162-0177		Western blot
Bio-Rad Mini-protean Gel 4–15%	Bio-Rad	456-1084		SDS-PAGE
Bio-Rad Mini-protean Gel 7.5%	Bio-Rad	465-1024		SDS-PAGE
4× Laemmli sample buffer	Bio-Rad	161-0747	1×	SDS-PAGE
Calphostin C	Sigma-Aldrich	C6303	1 μM	IDT uptake, MFZ 9-18 binding assay
Cytochalasin D	Sigma-Aldrich	C8273	2 μM	IDT uptake, MFZ 9-18 binding assay
PMA	Sigma-Aldrich	P8139	200 nM	IDT uptake, MFZ 9-18 binding assay
Monensin	Sigma-Aldrich	M5273	2.5 μM	IDT uptake, MFZ 9-18 binding assay
Nocodazole	Sigma-Aldrich	M1404	2 μM	IDT uptake, MFZ 9-18 binding assay
MFZ 9-18	Dr. Amy Newman Lab		100 nM	MFZ 9-18
LY333531	Sigma-Aldrich	SML0693	0.6 nM	IDT uptake, MFZ 9-18 binding assay
Bafilomycin A	Fisher Scientific	AAJ61835MCR	0.6 μM	IDT uptake, MFZ 9-18 binding assay
Dynasore	Sigma-Aldrich	D7683	80 μM	IDT uptake, MFZ 9-18 binding assay
Sodium dodecyl sulfate	Bio-Rad	1610302	10-1%	3H-DA uptake, Western blot
3H-DA uptake solution 1× (pH = 7.4)				
NaCl	Sigma-Aldrich	S9625	120 μM	
Tris Base	Bio-Rad	1610716	4 μM	
Hepes	Sigma-Aldrich	H4034	6.25 μM	
CaCl_2_ 2H_2_O	Sigma-Aldrich	C1016	2 M	
MgSO_4_ 7H_2_O	Sigma-Aldrich	230391	2 M	
KCl	Sigma-Aldrich	P3911	5 μM	
Ascorbic acid	Sigma-Aldrich	A92902	0.1 μM	
Ro410960	Sigma-Aldrich	R108	0.6 μM	
3H-DA stop solution (pH = 7.35)				
LiCl	Sigma-Aldrich	L9650	120 μM	
Tris base	Bio-Rad	1610716	4 μM	
Hepes	Sigma-Aldrich	H4034	6.25 μM	
CaCl_2_ 2H_2_O	Sigma-Aldrich	C1016	2 M	
MgSO_4_ 7H_2_O	Sigma-Aldrich	230391	2 M	
KCl	Sigma-Aldrich	P3911	5 μM	
IDT uptake solution (pH = 7.35)				
NaCl	Sigma-Aldrich	S9625	120 μM	
KCl	Sigma-Aldrich	P3911	1.3 μM	
CaCl_2_ 2H_2_O	Sigma-Aldrich	C1016	2.2 μM	
MgSO_4_ 7H_2_O	Sigma-Aldrich	230391	1.2 μM	
Hepes	Sigma-Aldrich	H4034	10 μM	
Glucose	Sigma-Aldrich	D9434	1 g/L	

DAPI, 4′,6-diamidino-2-phenylindole; DMEM, Dulbecco's modified Eagle's medium.

**Table 5:** Antibodies used for experiments

Description	Vendor	Catalog number	Concentration
DAT mouse mAb	Millipore	mAb 369	1:500
FLAG mouse mAb	Fisher	3165	1:200
mCh Rat mAb- 16D7	Invitrogen	M11217	1:500
RAB antibody panel	Cell Signaling Technology	9385T	1:200
Alexa 546 goat anti-Rat	Invitrogen	A21434	1:500
Alexa 488 goat anti-Rabbit	Invitrogen	A11034	1:500
Alexa 647 goat anti-mouse	Invitrogen	A21237	1:500
Novex Donkey anti-Rat HRP conjugated	Invitrogen	A18739	1:1000

### Plasmids and cloning

All plasmids were obtained from Addgene. The DAT was cloned into the Cry2–mCh plasmid to express Cry2–mCh at the N terminus of the DAT to ensure proper function because cloning of N-terminal–expressing YFP and FLAG to generate YFP–DAT and FLAG–DAT resulted in normal DAT function (12, 35, 38). The Cry2PHR–mCh (Addgene) vector was digested with BsrgI and NotI (New England Biolabs, Ipswich, MA). The DAT sequence was amplified from YFPsynDAT (Addgene), with flanking ends complementary to BsrgI (5′- TGA CCA TTG AAA CGG TAC CAC CAT GAG CAA GT-3′) and NotI (5′- TGC AAC TGC GGC CGC TCT CTT CAC ACC TTC AGC CAG T- 3′) sequences. DAT amplification products were isolated by gel electrophoresis and ligated into the digested Cry2–mCh vector using the Quick Ligation kit (New England Biolabs). The sequence was verified by sequencing and allele-specific polymerase chain reaction. Cry2–mCh–DAT (hereafter referred to as Cry2–DAT)–transformed DH5-alpha bacteria were cultured to propagate and isolate plasmids. We then isolated the plasmids by using Qiagen midiprep kit (Qiagen).

### Cell lines and cell culture

HEK293 cells and HEK293 cells stably expressing YFP–DAT (referred to as YFP–DAT cells) or FLAG–DAT (referred to as FLAG–DAT cells) were cultured in Dulbecco's modified Eagle's medium supplemented with 10% fetal bovine serum (FBS) (Atlanta Biologicals) and 1% penicillin/streptomycin (Gibco) at 37 °C and 5% CO_2_. SHSY-5Y cells were acquired from ATCC (ATCC) and cultured in Eagle's minimum essential medium/Ham's F-12 nutrient mixture medium (Gibco) and supplemented with 10% FBS (Atlanta Biologicals). To produce differentiated SHSY-5Y cells, they were treated with 10 μM retinoic acid (Sigma-Aldrich) for 7 days in EMEM with 0.5 to 1.0% FBS. The medium was refreshed the third day of culture (70).

HEK293 parental cells, HEK cells stably expressing YFP–DAT, or differentiated SHSY-5Y cells were transiently transfected with Cry2–DAT plasmid or Cry2–mCh empty vector using Lipofectamine 2000 (Invitrogen-Thermo Scientific) for 18 to 24 h. The medium was replaced with complete media after 24 h and used for experimentation 36 h after transfection.

### Western blot analysis of membrane-enriched DA and METH-induced YFP–DAT multimers

YFP–DAT HEK293 cells were seeded in tissue culture treated CELLSTAR dishes (Greiner Bio) and incubated at 37 °C and 5% CO_2_ overnight or until they reached 80 to 90% confluency. Cells were treated with the vehicle control, 10 μM METH, or 10 μM DA for 0.5, 2, 10, 30, or 60 min. After treatment, cells were washed with PBS. CuSO_4_ and 1,10-phenanthroline (Sigma-Aldrich), referred to as copper phenanthroline (100 μM CuSO_4_ and 400 μM 1,10-phenanthroline), in PBS with the protease inhibitor (PI) cocktail was added to cells for 10 min at room temperature (RT). Cross-linking was neutralized with 10 mM NEM for 20 min at RT. Cells were then scrapped into 1.7-ml conical tubes and centrifuged at 1200 rpm for 5 min to remove residual reagents and washed with PBS before membrane enrichment step. Membrane enrichment was performed using the Mem-PER Plus Membrane Protein Extraction kit (Thermo Scientific) according to the protocol. Membrane-enriched samples were quantified and diluted with 2× Laemmli Sample buffer (Bio-Rad) and incubated at RT for 30 min. Samples were not heated to maintain multimer complexes. Western blot was performed on samples to analyze the formation of multimers for each treatment.

### Blue light stimulation of Cry2–DAT multimers

The amount of blue light delivered to the cells was normalized throughout all experiments using a power meter to measure the amount of light output at the 20× and 60× objectives of widefield and confocal microscopes, respectively. On the Nikon TE2000E widefield microscope using a 20× with a 0.75 numerical aperture (NA) lens and an FITC HyQ filter from chroma, blue light output was measured at approximately 12.8 microwatts (μW) at the objective. The Nikon A1R confocal microscope at 4% of total output power of the 488 nm laser delivered 12.5 to 13 μW of light through a 60× Plan Apo 1.4 NA objective lens. Nikon Elements Advanced Research imaging software (Nikon instruments) was used for the automated exposure of blue-light pulses to the cells. To administer the blue light stimulation to cells using the widefield microscope, a 30-s pulse was delivered to the entire plate. In live-imaging experiments such as uptake, the 488-nm laser was set to 4% of total power output to deliver a 30-s pulse to the cells before initiating the experiment.

### Cell-surface biotinylation

HEK293 cells were seeded and transiently transfected with Cry2–DAT in 100-mm CELLSTAR dishes (MilliporeSigma) and incubated at 37 °C and 5% CO_2._ Cells were treated with the vehicle, 10 μM METH, or 10 μM NOM and then stimulated with 12.8 μW of blue light for 30 s or immediately washed with ice-cold PBS. Pulsed cells were incubated under red light conditions for 5 min before proceeding to the wash step. The cell-surface biotinylation protocol was followed as described (42). Cells were washed three times with ice-cold PBS and biotinylated at 4 °C for 45 min (1 mg/ml sulfosuccinimidyl-2-(biotinamido) ethyl-1,3-dithiopropionate) (EZ-Link Sulfo-NHS-S-S-biotin, Thermo Fisher Scientific). After the biotinylation step, the reaction was quenched with 100 mM glycine, and then, the cells were gently scraped into conical tubes and washed with PBS *via* centrifugation at 1500 rpm for 5 min at 4 °C. Cell pellets were solubilized in TNE buffer (150 mM Tris, 150 mM NaCl, 1 mM EDTA, 1% Triton X-100, pH 7.5) with PI (Thermo Fisher Scientific) for 2 h at 4 °C with periodic vortexing. Protein was isolated by centrifugation at 13,000*g* for 20 min at 4 °C. An aliquot of each sample was acquired for input analysis. Lysates were then incubated with 100 μl NeutrAvidin beads (Thermo Fisher Scientific) overnight at 4 °C. Samples were washed with chilled PBS + PI 3 times by centrifugation at 1200 rpm for 5 min. Biotinylated protein was extracted using Laemmli buffer with β-mercaptoethanol and heated at 95 °C for 5 min and then centrifuged to separate the beads from the biotinylated sample. To analyze proteins, samples were subjected to SDS-PAGE and transferred to membranes for Western blot analysis.

### Live-cell FRET in YFP–DAT– and Cry2–DAT–expressing HEK cells

FRET data were acquired according to Butler *et al.* (36). Briefly, HEK293 cells stably expressing YFP–DAT were plated on coverslips for 24 h before transient transfection with Cry2–DAT or CFP–DAT. Cells were treated with the vehicle or 10 μM METH for 2 min and then washed with PBS. Using a Nikon A1R confocal microscope with a 60× 1.4 NA oil-immersion lens, cells were stimulated with blue light (488 nm, 4% laser output), as indicated, immediately before beginning FRET acquisition. For cells coexpressing YFP–DAT and Cry2–DAT, the acceptor fluorophore (Cry2–DAT) was photobleached using a 560-nm laser for 0.5 s at 100% total laser output in a region of interest (ROI) placed at the PM. Similarly, for cells coexpressing YFP–DAT and CFP–DAT, the acceptor fluorophore (YFP–DAT), was photobleached with 514-nm laser for 0.5 s with 100% total laser output in an ROI placed at the PM. For cells coexpressing YFP–DAT and Cry2–DAT, the donor fluorophore, YFP–DAT, was imaged using a 488-nm laser excitation and 500- to 550-nm emission filter. Measurements for YFP–DAT were acquired for 2 min. Similarly, for cells coexpressing YFP–DAT and CFP–DAT, the donor fluorophore, CFP–DAT, was imaged using a 457-nm laser excitation and 464- to 499-nm emission filter. The data were exported from NIS-Elements to Excel spreadsheets. The background was subtracted from prebleach and postbleach time points. FRET efficiencies were calculated according to Equation 1.

(1) $${\text{FRET}}_{\text{eff}}=\frac{\left({\text{donor}}_{\text{post}}-{\text{BkGrnd}}_{\text{post}}\right)-\left({\text{donor}}_{\text{pre}}-{\text{BkGrnd}}_{\text{pre}}\right)}{\left({\text{donor}}_{\text{post}}-{\text{BkGrnd}}_{\text{post}}\right)}\text{×}100$$

### Live-cell fluorescence recovery after photobleaching (FRAP) analysis of YFP–DAT and Cry2–DAT

HEK293 cells stably expressing YFP–DAT were plated on coverslips and transiently transfected with Cry2–DAT 24 h after plating. Cells were treated with the vehicle or 10 μM METH for 2 min and then washed with PBS. Cells were exposed to 4% 488-nm light for 30 s, as indicated, immediately before beginning FRAP data acquisition using a Nikon A1R confocal microscope equipped with 60× 1.4 NA oil-immersion objective lens and stage-top incubator. A 5-μm circular ROI at the PM was selected for FRAP. Three prebleach images were acquired before photobleaching the ROI with 488 nm (YFP–DAT) or 560 nm (Cry2–DAT) for 0.5 s at 100% total power output. Recovery of fluorescent molecules into the photobleached ROI was acquired at 1.5% laser power for 300 s. The raw data were exported using Nikon Elements software to Microsoft Excel spreadsheets. For each experiment, the background fluorescence was subtracted, and the data were normalized to the whole-cell fluorescence to assure normalization across cells with various levels of expression. The normalized FRAP recovery curves were fitted to a one-phase exponential association function using GraphPad Prism 8, and curves with R^2^ < 0.9 were excluded. The D and M_f_ were calculated using the following equations:

(2) $$\text{D}=0.224{\text{r}}^{2}/{\text{t}}_{1/2}$$

where D is the diffusion coefficient (μm^2^/s), r is the bleach radius (μm), and t_1/2_ is the half time of recovery (s).

(3) $${\text{M}}_{\text{f}}=\left({\text{F}}_{\infty }-{\text{F}}_{0}\right)/\left({\text{F}}_{\text{i}}-{\text{F}}_{0}\right)\times 100$$

where M_f_ is the mobile fraction (%), F_∞_ is the average of the last 5 data points of the experiment, F_0_ is the first data point after the photobleach, and F_i_ is the average of the prebleach intensity (59).

### DAT-mediated uptake by IDT307 (APP^+^)

HEK293 cells stably expressing FLAG–DAT or transiently transfected with Cry2–DAT were seeded into 35-mm glass-bottom MatTek dishes (MatTek). For each Cry2–DAT experiment, FLAG–DAT uptake under control conditions was used as a positive control and normalization of data. FLAG–DAT and Cry2–DAT activity was examined using IDT307–[4-(4-(dimethylamino)phenyl)-1-methylpyridinium iodide]. IDT307 is a fluorescent neurotransmitter substrate used to test the real-time uptake using fluorescence microscopy (13, 43). It is nonfluorescent in solution, but fluoresces as it binds to the DAT and accumulates in the cytoplasm. It was reconstituted as described (71). Cells were treated with the vehicle, 10 μM METH, or 10 μM NOM for 2 min before adding IDT307. For trafficking experiments, 1 μM Cal C, 2 μM cytochalasin D, 2 μM nocodazole, or 2.5 μM monensin was used to treat the cells before treatment with vehicle or METH. The cells were then washed twice with IDT uptake buffer, and 750-μl fresh IDT buffer was added to the plate. Cells were then imaged with a Nikon A1R confocal laser scanning microscope using a 60× 1.4 NA oil-immersion objective and excitation/emission wavelengths of 488/500 to 550 nm for green fluorescence (and blue light stimulation) and 561/570 to 620 nm for red fluorescence. For conditions of Cry2–DAT multimerization, cells were stimulated with 488-nm laser set to 4% of the total power output to deliver a 30-s pulse automatically using NIS software immediately before performing uptake. For each experiment, 30 s was allotted to establish a baseline before the addition of IDT. At 30 s, 250 μl IDT307 in IDT uptake buffer was added to the side of the plate to make a final concentration of 10 μM IDT307. The total imaging time was 6 min.

Data were pooled from three separate experiments, and a minimum of five cells per experiment were evaluated. Changes in cellular IDT307 uptake were measured using Nikon Elements Advanced Research analysis software. The transport of IDT307 into the cells was determined as average fluorescence units in the selected ROI of each cell using the Nikon Elements software. The average fluorescence units throughout the time of the experiment were recorded for each cell and exported into an Excel spreadsheet. For Cry2–DAT analysis, parental nontransfected cells were analyzed separately from Cry2–DAT cells and served as an internal control for the transfected experiments. The background of each experiment was subtracted and then normalized to the baseline average (time lapse) or to FLAG–DAT control (total uptake). Fluorescence measurements are shown as the fold change (baseline normalization) or as fold change in respect to FLAG–DAT control experiment. Statistical relevance (*p* < 0.05) was determined by one-way ANOVA with Tukey’s multiple comparisons tests in GraphPad Prism software.

### [^3^H]DA-uptake assay

HEK293 parental cells stably expressing FLAG–DAT were seeded in 24-well dishes, previously coated with poly l-lysine at a density of 50,000 cells/well. HEK parental cells were transfected with Cry2–DAT plasmid as previously described. The [^3^H]DA-uptake assay was performed as previously outlined (42). Saturation assays were performed using dilutions of 2 μM, 200 nM, 20 nM, 2 nM, and 0.2 nM, and the control was prepared in the uptake buffer (4 mM Tris, 6.25 mM HEPES, 120 mM NaCl and 5 mM KCl, pH 7.4 with HCl). Nonspecific uptake was measured in the presence of 26.4 μM cold DA. Cells were treated with the dilutions, and uptake was facilitated for 5 min at 37 °C. For the other experiments, control, 10 μM METH, or 10 μM NOM was pretreated. Cells were pulsed in plates for 30 s on the Nikon TE 2000 widefield microscope. Uptake was performed using 20 nM [^3^H]DA for 5 min at 37 °C for single-dose experiments. After uptake, all contents in the well were removed and replaced with the wash buffer (4 mM Tris, 6.25 mM Hepes, and 120 mM LiCl) to stop the reaction. The wash buffer was removed from the wells, and 1% SDS was added to cells and incubated on a plate rocker for 1 h. The entire content of each cell was transferred to scintillation tubes containing Bio-Safe II Complete Counting Cocktail (RPI-111195, Research Products International). The radioactivity of the samples was measured using a Liquid Scintillation Counter model LS6500 (Beckman Coulter). NSP readings were subtracted from readings for each condition along with background to assess specific activity of each condition. Saturation assays were performed in quadruplets, and the treatment paradigm was performed in sextuplets. To assess statistical changes, we performed one-way ANOVA with post hoc Tukey's test.

### MFZ 9-18 binding assay in FLAG–DAT– and Cry2–DAT–expressing cells

MFZ 9-18 is a fluorescent COC analog used to visualize the DAT at the PM of DAT-expressing cells (44). MFZ 9-18 was synthesized using previously published methods (44).

FLAG–DAT cells or HEK293 cells transiently transfected with Cry2–DAT were seeded on coverslips. Cells were washed with PBS before treatments with 10 μM METH or NOM in PBS for 30 min at 37 °C. For trafficking experiments, 1 μM Cal C, 2 μM cytochalasin D, 2 μM nocodazole, or 2.5 μM monensin was used to treat the cells before treatment with METH. After treatment, where indicated, cells were pulsed with blue light using a Nikon TE2000E widefield microscope (20× 0.75 NA objective lens) for 30 s using the FITC HyQ filter from Chroma as previously described. After the blue light pulse, the cells were incubated for 5.5 min to mimic the IDT307 uptake protocol times. The buffer was removed, and ice-cold MFZ 9-18 (100 nM) was gently added to the plates. The cells were incubated with MFZ 9-18 at 4 °C for 15 min before imaging. Using a 488-nm laser on a Nikon A1R confocal, cells were imaged immediately after MFZ 9-18 incubation. For each image, ROIs were drawn at the PM of the cells. The MFZ 9-18 fluorescence intensity was measured for each ROI, and the data were exported into Excel spreadsheets. The background was subtracted from each image and normalized to parental MFZ 9-18 binding. Data were analyzed for statistical significance using GraphPad Prism 8.

### Immunocytochemistry to determine DAT subcellular localization

HEK293 parental cells were plated on coverslips and transiently transfected with Cry2–DAT plasmid. HEK cells stably expressing FLAG–DAT were also seeded on coverslips. At the appropriate density and protein expression, cells were treated with the vehicle control or 10 μM METH for 2 min, followed by blue light stimulation, as indicated. Cells were then fixed using 3.7% paraformaldehyde for 15 min at RT. Cells were blocked with the permeabilization and blocking buffer (5% FBS, 0.1% Triton-X 100, and PI in PBS) for 30 min at RT. Coverslips were then incubated in primary antibodies diluted in the permeabilization and blocking buffer (FLAG mAb, 1:500 [Fisher, Cat No. 3165], mCh mAb1:100 [Invitrogen, M11217], and Rab antibody panel 1:500 [Cell Signaling Technology, Cat No. 9385T]) for 30 min. Coverslips were washed 3 times for 5 min with PBS containing PI, and then incubated in Alexa Fluor 488 donkey anti-rabbit for Rab 4, 5, 7, or 11 (Invitrogen, Cat No. A11034), Alexa Fluor 546 goat anti-rat for Cry2-mCh–DAT (Invitrogen, Cat No. A21434), and Alexa Fluor 647 goat anti-mouse (Invitrogen, Cat No. A21237) for FLAG–DAT for 30 min at RT. Coverslips were washed before mounting onto slides with Diamond antifade mounting media with 4′,6-diamidino-2-phenylindole (Thermo Fisher Scientific) and dried overnight at RT in the dark. Imaging was performed using a Nikon A1R confocal laser scanning microscope using excitation/emission wavelengths of 488/500 to 550 nm for green fluorescence, 561/570 to 620 for red fluorescence, and 647/640 to 680 nm for far-red fluorescence. ROIs were drawn at areas of Rab puncta to determine colocalization with Cry2–DAT *via* Pearson’s correlation coefficients using Nikon Elements Advanced Research imaging software.

### Statistical analysis

All data are shown as the mean ± SE unless otherwise noted. For colocalization measurements, Nikon Elements Advanced Research imaging software was used to determine Pearson’s correlation coefficient. Data were analyzed using a Student's *t* test or one-way ANOVA with Tukey’s multiple comparisons test for post hoc analysis. Data are considered statistically relevant when *p* < 0.05. Statistical analysis was run on GraphPad Prism 9.0 (GraphPad Software). The specific tests used to determine significance for each figure are described in the figure legends.

## Data availability

All relevant data are contained within the article and supporting information.

## Supporting information

This article contains supporting information.

## Conflict of interest

The authors declare that they have no conflicts of interest with the contents of this article.
