# Supplementary material for: Optogenetically-induced multimerization of the dopamine transporter increases uptake and trafficking to the plasma membrane
Source: J Biol Chem. 2021 May 18;296:100787. doi: 10.1016/j.jbc.2021.100787 (PMC8203837; doi:10.1016/j.jbc.2021.100787)
Supplement: Figures S1–S4 [file mmc1.pdf]

Supplemental Figures

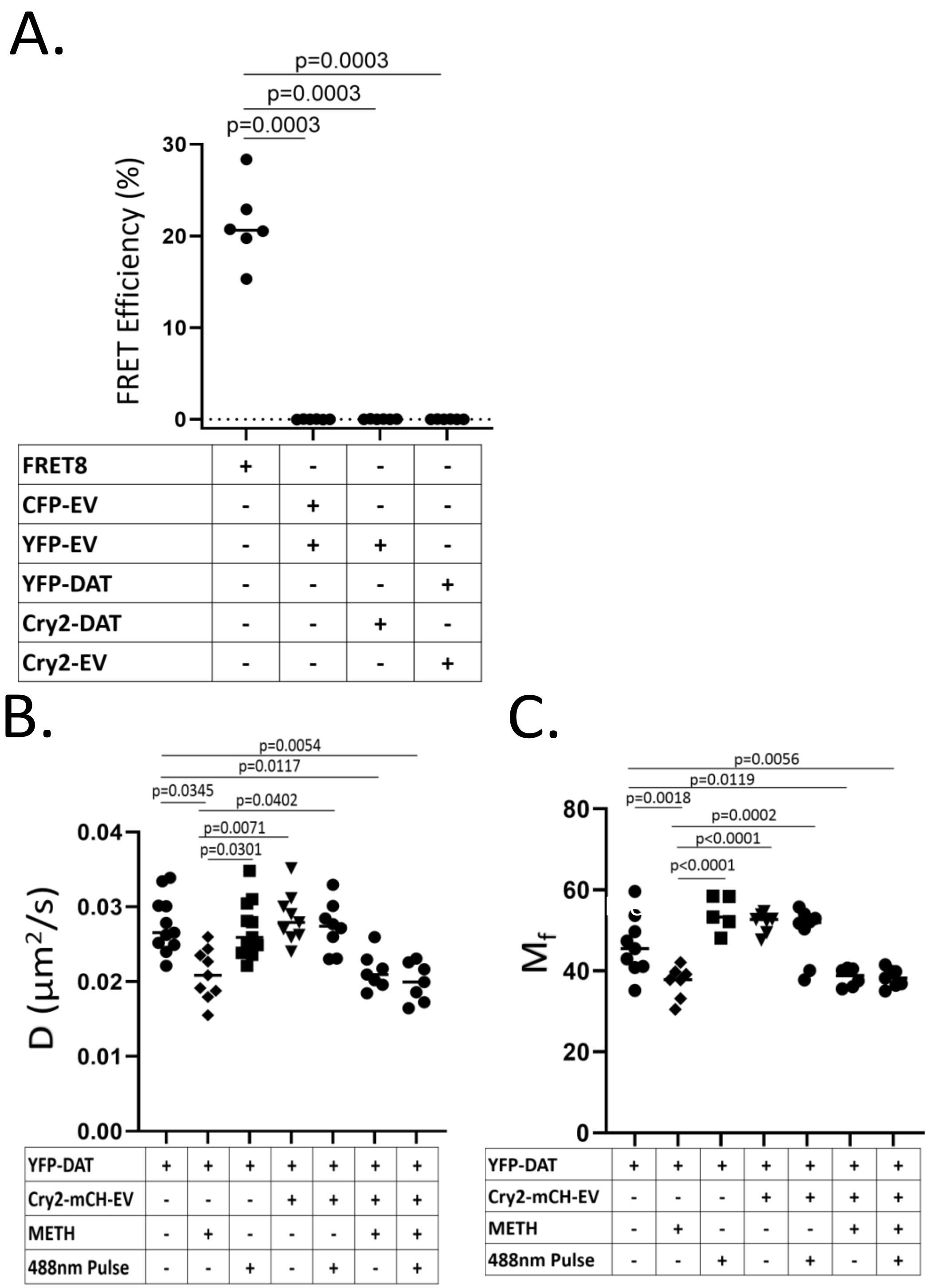

Figure S1. A) FRET8 (CFP and YFP tethered by an 8 amino acid linker) and YFP-empty vector (EV), CFP-EV, YFP-DAT, Cry2-mCh-EV and CRY2-DAT were tested for FRET efficiency to confirm the non-

specificity of the constructs (n=6, P=0.0003). B) FRAP controls to test D and Mf of YFP-DAT alone and YFP-DAT and Cry2-mCh-EV co-expressed to test if the empty vector affects YFP-DAT D and Mf (n=7-9, 0.001>p<0.05).

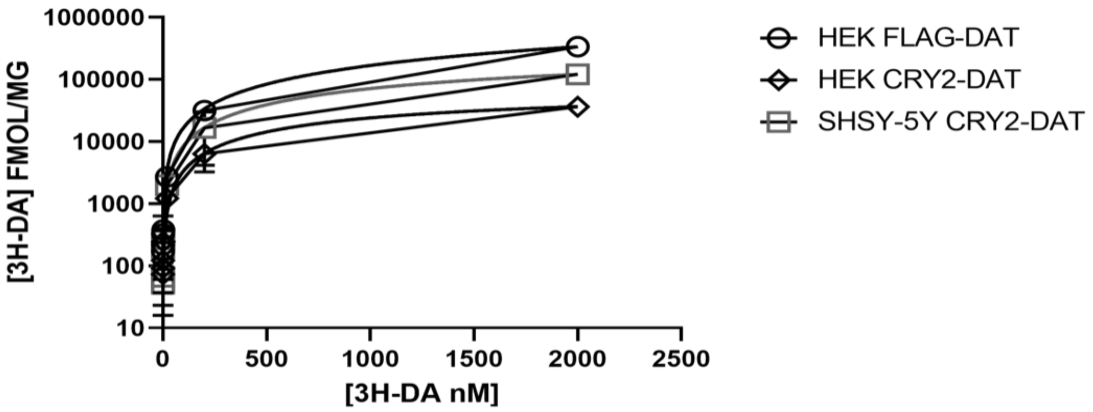

	HEK FLAG-DAT	HEK CRY2-DAT	SHSY-5Y CRY2-DAT
Bmax	3117511	117999	299886
Kd	17899	2513	2121
R Squared	0.9954	0.9615	0.9885

Figure S2. 3H-DA uptake saturation in HEK FLAG-DAT, HEK Cry2-DAT and SHSY-5Y Cry2-DAT cells performed to demonstrate kinetics of the assay in each cell line under control conditions. Bmax, Kd and R squared values for each are listed below.

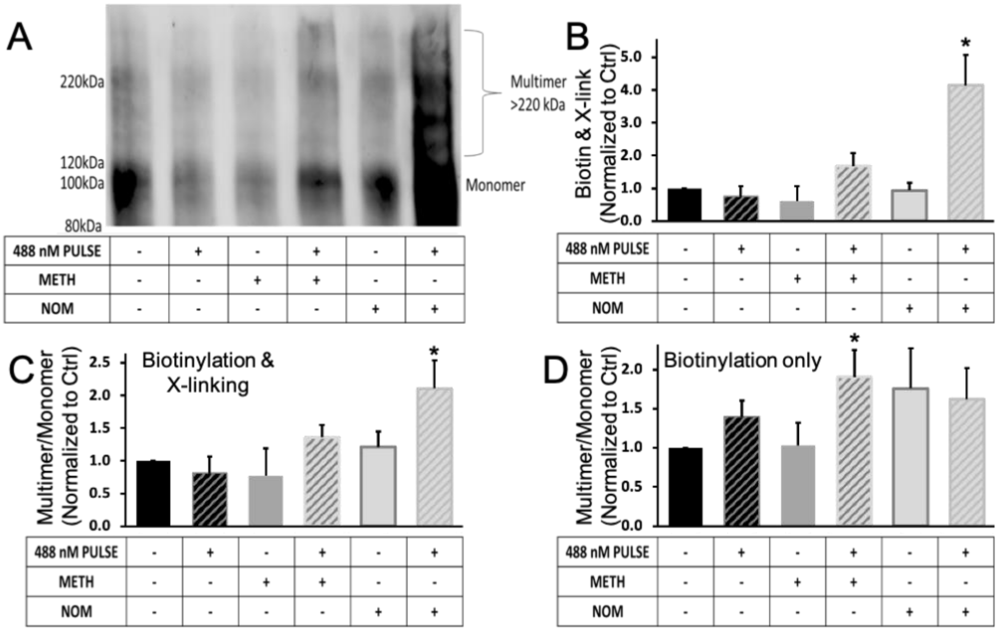

Figure S3. CuP crosslinked cell surface biotinylation was done to examine Cry2-DAT multimers at the cell surface. A) Representative blot of CuP crosslinked cell surface biotinylated Cry2-DAT. B) Analysis of immunoreactivity of biotinylated Cry2-DAT western blots in A show a statistically significant increase in

cell surface DAT with 10 µM NOM treatment and subsequent 488 nm light pulse (n=3). Cry2-DAT multimer to monomer ratio was calculated and normalized to control for C) blots showing cross-linked and biotinylation as shown in A and D) blots with biotinylation only as shown in Figure 5C. The asterisk indicates p<0.05.

A.

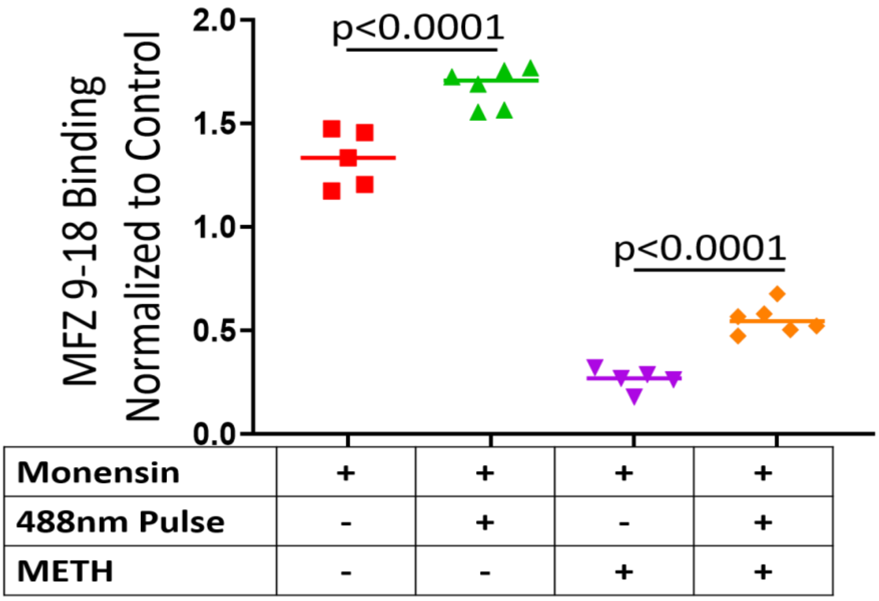

B.

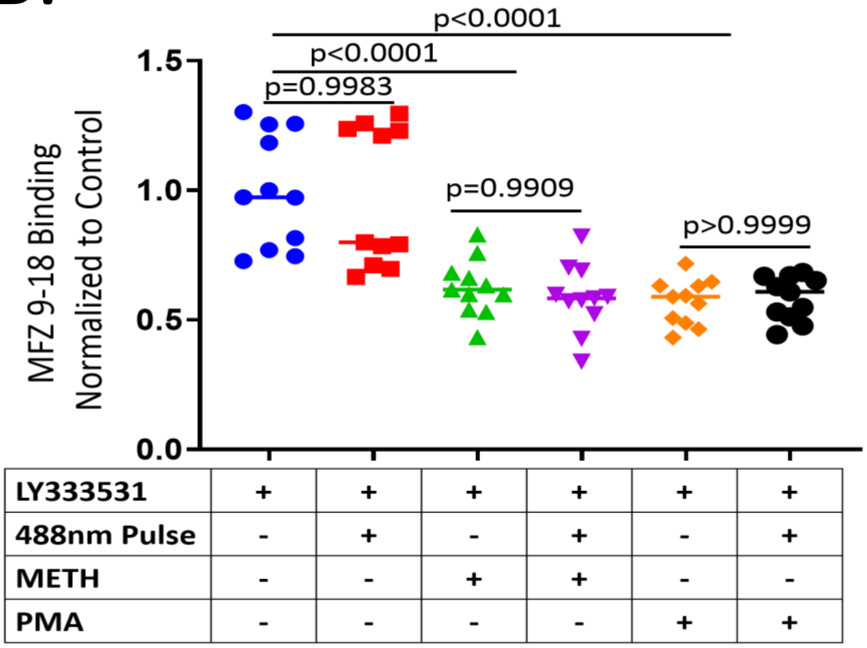

Figure S4. A) HEK cells were transfected with Cry2-DAT, and MFZ 9-18 binding was measured after pretreatment with 2.5 µM monensin, 10 µM METH, 10 µM NOM, or exposed to 488 nm light for 30s where indicated. Monensin did not inhibit the increase in MFZ 9-18 binding upon blue light stimulation. (n=4-6, 0.0001<p>0.0065). B) HEK cells were transfected with Cry2-DAT, and MFZ 9-18 binding was measured after pretreatment with 2 µM nocodazole, 10 µM METH, 10 µM NOM, or exposed to 488 nm light for 30s where indicated. Nocodazole did not inhibit the increase in MFZ 9-18 binding upon blue light stimulation. (n=15, 0.0001>p<0.9994).
